# Dual-purposing disulfiram for enhanced chemotherapy and afterglow imaging using chlorin e6 and semiconducting polymer combined strategy

**DOI:** 10.7150/thno.96136

**Published:** 2024-08-19

**Authors:** Di Zhao, Aifang Zhou, Xintong Dong, Hong-Min Meng, Yating He, Lingbo Qu, Ke Zhang, Yuehe Lin, Zhaohui Li

**Affiliations:** 1 College of Chemistry, Henan Joint International Research Laboratory of Green Construction of Functional Molecules and Their Bioanalytical Applications, Zhengzhou University, Zhengzhou 450001, P. R. China.; 2 Department of Chemistry and Chemical Biology, Northeastern University, Boston, Massachusetts 02115, United States.; 3 Department of Chemistry, School of Mechanical and Materials Engineering, Washington State University, Pullman, Washington 99164, United States.

**Keywords:** chemotherapy, disulfiram, chlorin e6, semiconducting polymer nanocomplex, afterglow imaging

## Abstract

**Rationale:** One of the main challenges in chemotherapy is achieving high treatment efficacy while minimizing adverse events. Fully exploiting the therapeutic potential of an old drug and monitoring its effects *in vivo* is highly valuable, but often difficult to achieve.

**Methods:** In this study, by encapsulating disulfiram (DSF) approved by US Food and Drug Administration, semiconducting polymer nanocomplex (MEHPPV), and Chlorin e6 into a polymeric matrix F127 via nanoprecipitation method, a nanosystem (FCMC) was developed for afterglow imaging guided cancer treatment. The characteristics, stability as well as the ability of singlet oxygen (^1^O_2_) production of FCMC were first carefully examined. Then, we studied the mechanism for enhanced anti-cancer efficiency and afterglow luminescence *in vitro*. For experiments* in vivo*, 4T1 subcutaneous xenograft tumor mice were injected with FCMC via the tail vein every three days and the antitumor effect of FCMC was evaluated by monitoring tumor volume and body weight every three day.

**Results:** The nanosystem, which combines DSF with Ce6, can generates abundant ^1^O_2_ that enhances the antitumor activity of DSF. The *in vivo* results show that FCMC-treated group exhibits an obviously higher tumor-growth inhibition rate of 67.89% after 15 days of treatment, compared to the control group of F127@MEHPPV-CuET. Moreover, Ce6 also greatly enhances the afterglow luminescence intensity of MEHPPV and promotes the redshift of the afterglow emission towards the ideal near-infrared imaging window, thereby enabling efficient afterglow tumor imaging *in vivo*.

**Conclusions:** This multifunctional nanoplatform not only improves the anticancer efficacy of DSF, but also enables monitoring tumor via robust afterglow imaging, thus exhibiting great potential for cancer therapy and early therapeutic outcome prediction.

## Introduction

Cancer remains a major health threat and chemotherapy is a well-established treatment strategy 1. However, conventional chemotherapy methods often fail to achieve satisfactory therapeutic outcomes due to low efficacy and nonspecific toxicity 2-4. Additionally, separate methods are typically needed to monitor treatment outcomes 5, 6. Therefore, there is an urgent need to improve treatment efficacy and establish easy-to-use methods to accurately monitor real-time therapeutic effects *in vivo*. Repurposing established chemotherapeutics with known antitumor mechanisms and pharmacology offers a quick and efficient strategy to achieve this goal 7-9.

Disulfiram (DSF) is used to treat alcohol addiction and has chemotherapeutic activity due to the ability to chelate with cupric ions (Cu^2+^) to form toxic CuET 10-12. CuET disrupts the p97-NPL4-UFD1 pathway and induces cell death by releasing Cu^2+^ under oxidative conditions 13-15. Although several groups have developed drug delivery systems for co-delivering DSF and Cu^2+^ for cancer therapy 16-18, one challenge remains: the inadequate reactive oxidation species (ROS) content in cancer cells plays an inhibitory role in triggering the efficient release of Cu^2+^ from CuET, thus resulting in insufficient antitumor activity. Combining CuET agents with ROS generators may become an effective approach for improving the treatment efficacy of DSF.

In addition, accurately imaging tumor *in vivo* via molecule imaging is crucial for predicting therapeutic efficacy and identifying off-target toxicity 19-22. Among the available imaging techniques, afterglow imaging shows great potential due to its high signal-to-background ratio (SBR) and deeper tissue penetration 23-25. In recent years, both inorganic and organic materials have been developed for afterglow imaging applications 26-28. Among them, organic afterglow nanoparticles based on poly[para-phenylenevinylene] (PPV), such as poly[2-methoxy-5-(2-ethylhexyloxy)-1,4-phenylenevinylene] (MEHPPV), have become increasingly attractive for biomedical imaging owing to their superior biocompatibility and biodegradability 29. Moreover, the afterglow intensity of these organic nanoparticles can be boosted by singlet oxygen (^1^O_2_), thereby increasing bioimaging sensitivity.

**Scheme 1:**
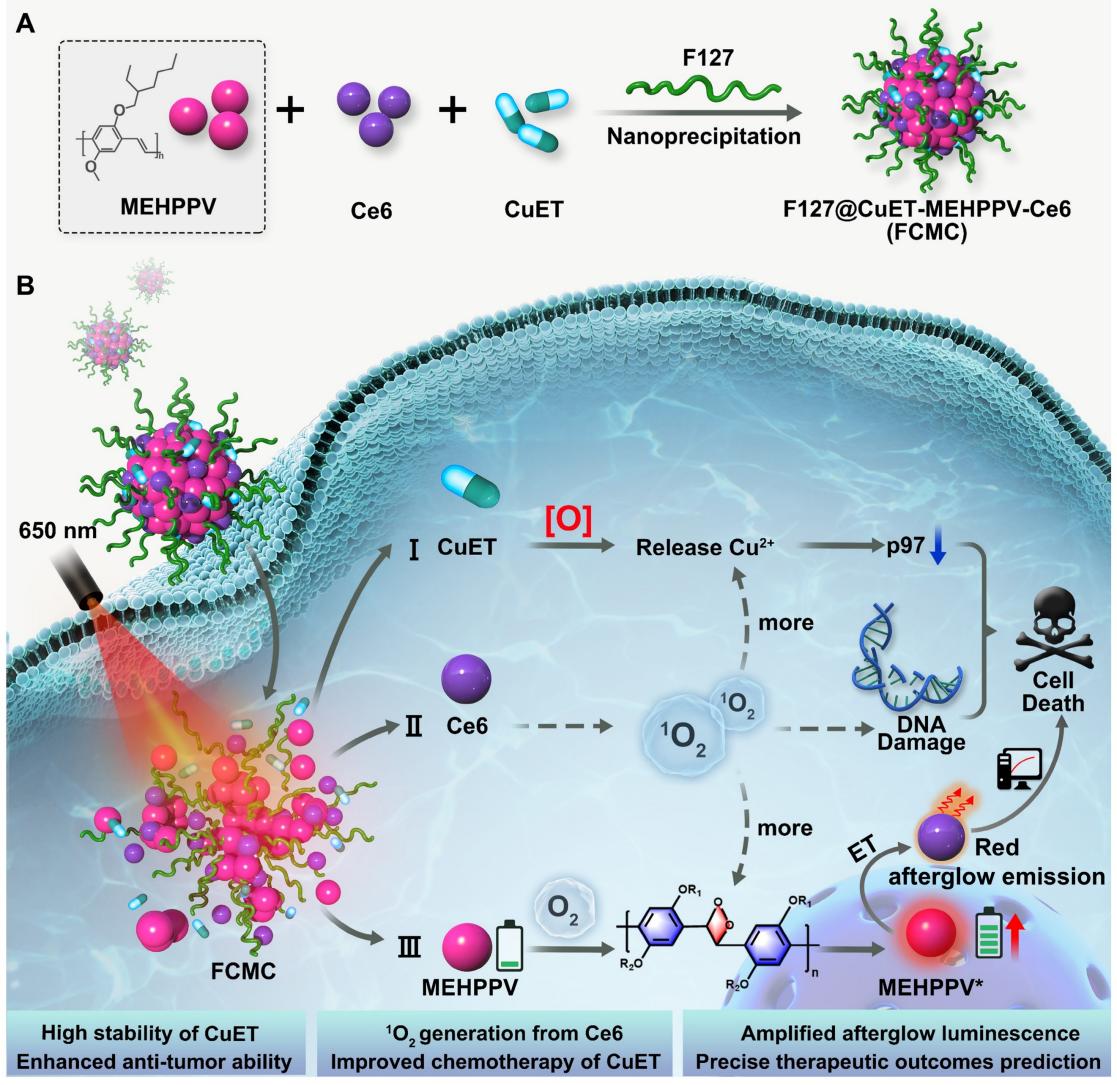
Schematic diagram of (**A**) the preparation of FCMC nanoparticle through nanoprecipitation strategy. (**B**) FCMC-induced antitumor activity and afterglow imaging process.

In this study, a simple and multifunctional semiconducting polymer nanocomplex, F127@CuET-MEHPPV-Ce6 (FCMC), was developed for cancer therapy (Scheme 1). By simultaneously encapsulating DSF-copper complexes (CuET), MEHPPV, and Ce6 into a polymeric matrix F127, this nanosystem facilitates simultaneously cancer therapy and afterglow imaging *in vivo*. We hypothesize that this nanoplatform works as follows: 1) Ce6 generates ^1^O_2_, which enhances Cu^2+^ release from CuET and improves DSF anticancer efficiency; 2) Ce6-doping amplifies the afterglow luminescence signal of MEHPPV as more ^1^O_2_ generated and promotes the redshift of the afterglow emission towards the ideal near-infrared (NIR) imaging window, providing high ratio of signal to background of tumor imaging with enhanced sensitivity; 3) CuET formed by Cu-S chelation of DSF and Cu^2+^ is more stable than free DSF in physiological environment, leading to a further improvement in therapeutic outcome. Therefore, this nanoplatform facilitates dual improvement of DSF anticancer efficiency, making it a promising strategy for cancer therapy.

## Results

### Synthesis and characterization of FCMC

**Figure 1:**
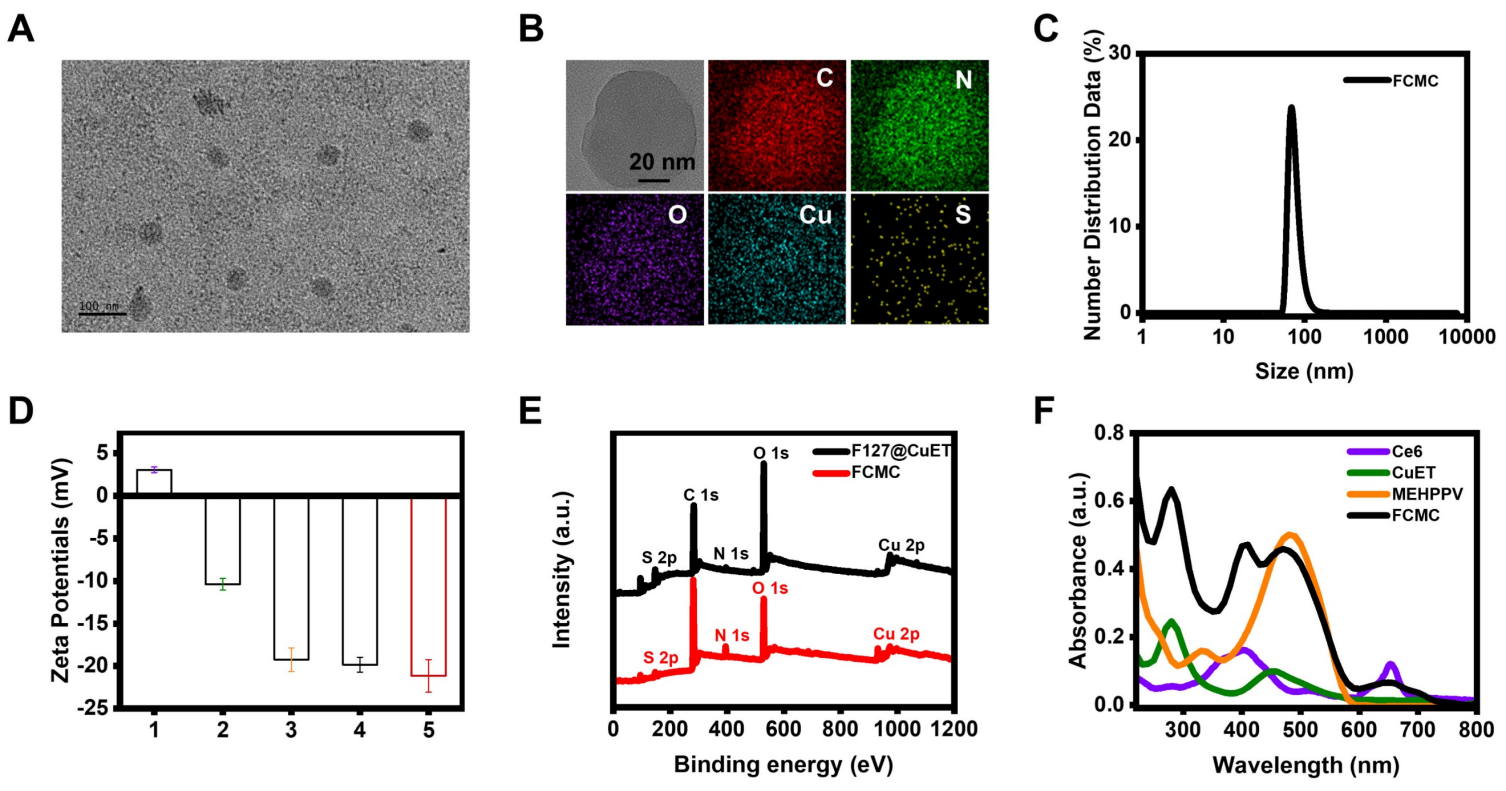
** FCMC characterization.** (**A**) TEM image of FCMC nanoparticles. (**B**) STEM images and corresponding elemental mapping of FCMC. (**C**) Dynamic light scattering of FCMC. (**D**) Zeta potentials of (1) CuET, (2) F127@CuET, (3) F127@Ce6, (4) F127@MEHPPV, and (5) FCMC. (**E**) XPS survey spectra of F127@CuET and FCMC. (**F**) UV-Vis absorbance spectra of Ce6, CuET, MEHPPV, and FCMC.

Nanoprecipitation was used to synthesize FCMC (Scheme. 1a) 30. CuET nanoparticles were first prepared using a coordinated self-assembly strategy with minor modifications (Figure S1) 31-33. The mechanism for preparation of CuET involves the following two steps: 1) the reaction between Cu^2+^ and DSF generates Bitt-4^2+^ intermediates and Cu^+^, 2) another DSF molecule chelates with two Cu^+^ ions to obtain one CuET complex and one Cu^2+^ ion 34, 35. Subsequently, CuET combined with F127, MEHPPV, and Ce6 to form FCMC. Characterization of FCMC using transmission electron microscopy (TEM) indicated that FCMC exhibits a uniform diameter of approximately 70 nm (Figure 1A). Moreover, scanning transmission electron microscopy (STEM) images and corresponding STEM-EDS elemental mapping revealed uniform C, N, O, Cu, and S distribution (Figure 1B). The hydrodynamic size of FCMC was approximately 80 nm with a polydispersity index of 0.228 (Figure 1C). Successful modification of F127 was indicated by the decreased zeta potential from +3.04 mV for CuET to -22.5 mV for FCMC (Figure 1D). In this work, different control nanoparticles were also synthesized and their hydrodynamic sizes as well as zeta potential were assessed (Figure 1D and Figure S2). Furthermore, X-ray photoelectron spectroscopy (XPS) survey spectra of FCMC showed obvious peaks for O 1s, S 2p, and Cu 2p (Figure 1E), and the binding energy centers at 930.2 eV and 950.5 eV were Cu 2p3/2 and Cu 2p1/2 satellite peaks, respectively (Figure S3). Moreover, typical UV-Vis absorbance peaks at approximately 275 nm, 400 nm, 500 nm, and 660 nm, also confirmed the successful preparation of FCMC (Figure 1F).

### FCMC stability in buffer and serum

As an essential parameter for FCMC performance, the stability of FCMC in buffer and serum was investigated. FCMC was incubated in phosphate buffered saline (PBS) for 1 h under different conditions [pH 5.4, pH 6.5, pH 7.4, 1 mM glutathione (GSH)], and the free Cu content was measured using Inductive Coupled Plasma Mass Spectrometer (ICP-MS). The released DSF in the solution was measured using UV-Vis spectral analysis, which indicated that the release efficiency of Cu and DSF were both less than 10% (Figures S4 and S5). Further investigation on the stability of FCMC by monitoring the change in particle size revealed no measurable swelling and the particle size remained stable in PBS with varying pH values, Dulbecco's Modified Eagle Medium (DMEM) containing 10% fetal bovine serum (FBS), as well as fetal bovine serum (FBS) at 37°C for 5 days (Figure S6). In addition, the effect of organic polymer compounds (such as, Triton X-100) and small molecule organic compounds (such as, urea) on the micellization of F127 was also investigated. The results were shown in Figure S7A, which indicated that as the proportion of Triton X-100 in solution increased, the particle size of FCMC gradually decreased. The addition of Triton X-100 may be beneficial for the hydrophobic core of F127 to encapsulate CuET, MEHPPV, and Ce6, while also increase the hydrophilicity of FCMC. However, there was almost no significant change in the DLS size of FCMC in the presence of urea, indicating that the effect of urea on F127 micellization was very limited (Figure S7B). These results demonstrated that FCMC was stable and could prevent DSF degradation during delivery.

### Enhanced afterglow luminescence with Ce6 doping

We hypothesized that ^1^O_2_ produced by the photosensitizer Ce6 could further activate MEHPPV to amplify afterglow luminescence. In this regard, a series of doping ratios of Ce6 was introduced to prepare FCMC with a fixed amount for MEHPPV (mMEHPPV: mCe6 = 1:0, 1:0.5, 1:1, 1:2, and 1:3). The loading rates of CuET, MEHPPV, and Ce6 in the synthesized FCMC at different doping ratios were measured using UV-Vis absorption spectroscopy, as shown in Table S1. Results from these investigations showed that the highest afterglow intensity was obtained when the mMEHPPV/mCe6 ratio was 1:1 (Figure S8), and a distinct afterglow signal could be observed throughout the 35-min decay period after irradiation. Under optimized conditions, the afterglow intensity of FCMC was much higher than that of F127@CuET-MEHPPV at the same MEHPPV concentration, which suggested that Ce6 contributed significantly to the overall afterglow luminescence intensity. Moreover, we found that 300 mW/cm^2^ laser power induced the brightest afterglow luminescence (Figure S9), which was selected for subsequent experiments.

**Figure 2:**
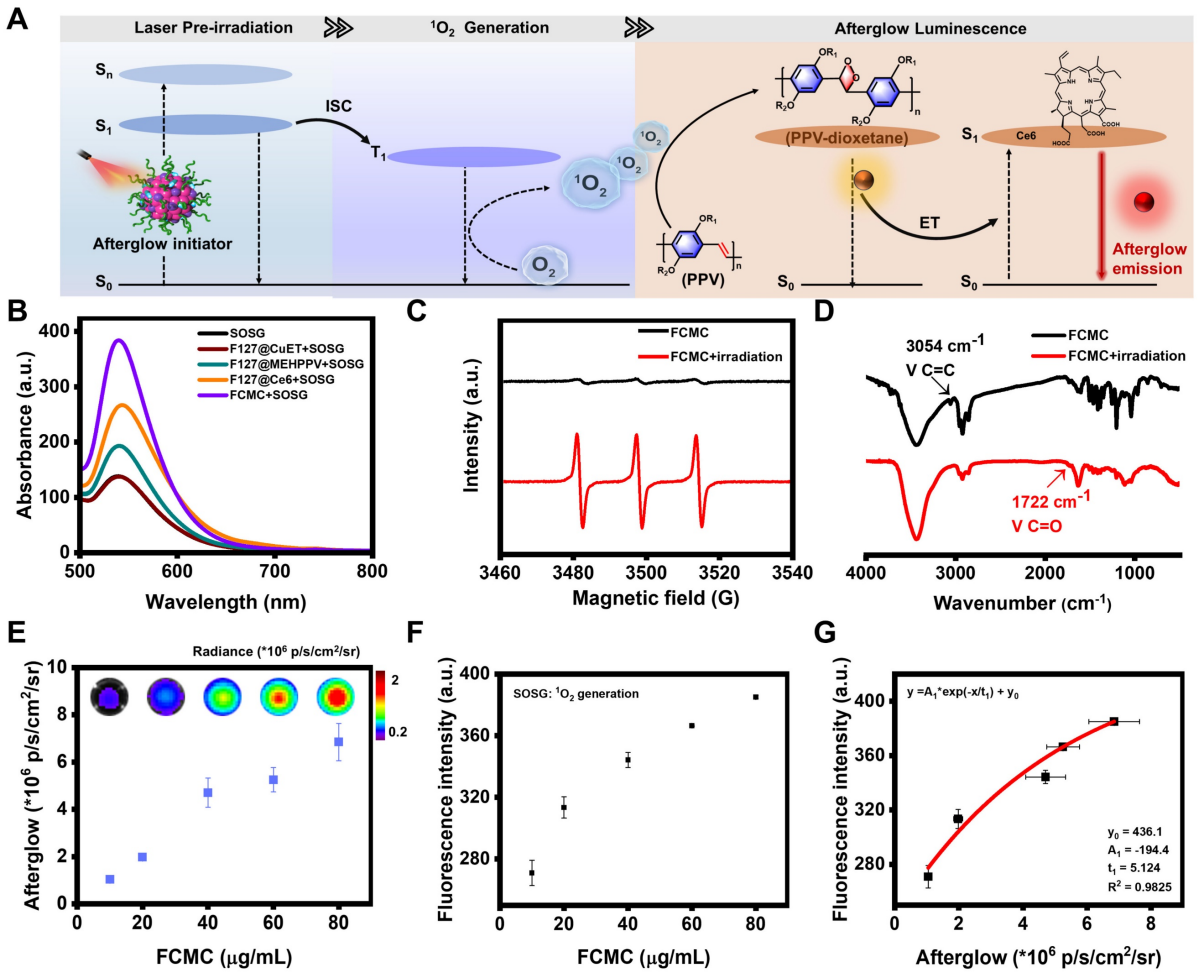
**Investigation of the afterglow luminescence mechanism.** (**A**) Schematic diagram of the afterglow luminescence mechanism of FCMC and Ce6-enhanced afterglow luminescence in FCMC. (**B**) Fluorescence spectra of SOSG: ^1^O_2_ generation capacity of different nanoparticles after 5 min of 650 nm laser irradiation. (**C**) EPR spectra of FCMC before or after 5 min of 650 nm laser irradiation. (**D**) FTIR spectra of FCMC before or after laser irradiation. (**E**) Afterglow images at different concentrations of FCMC and statistics of afterglow signal intensity. (**F**) ^1^O_2_ yield from FCMC at different concentrations after 5 min of light exposure. (**G**) Correlation between afterglow intensity and ^1^O_2_ yield of FCMC.

Here, we hypothesized that Ce6 played three synergistic roles in enhancing afterglow luminescence (Figure 2A): (1) absorbing and converting light energy into an ^1^O_2_ initiator 36, 37. (2) Promoting additional ^1^O_2_ and PPV-dioxetanedione (high-energy intermediate) generation, which can increase afterglow luminescence Intensity 38. (3) Acting as an energy acceptor, enabling NIR afterglow window imaging with a higher SBR through energy transfer (ET). To demonstrate our deduction, a series of experiments were carried out. The fluorescent intensity of singlet oxygen sensor green (SOSG, an ^1^O_2_ indicator) incubated with FCMC was much higher than that of the control groups (Figure 2B), indicating that Ce6 can promote ^1^O_2_ generation. Electron paramagnetic resonance (EPR) spectroscopy was performed to verify ^1^O_2_ production by using 2,2,6,6,-tetramethyl-4-piperidone (TEMP) as a spin trapping trap. The results indicated that FCMC produced the typical 1:1:1 peak signal under irradiation, which means FCMC has the capability to generate^ 1^O_2_ (Figure 2C). Moreover, the typical absorption peak of MEHPPV at 500 nm exhibited a significant decrease after illumination (Figure S10), indicating that the conjugated structure of MEHPPV in FCMC had been eliminated. Fourier transform infrared (FITR) spectrometer analysis showed that the ethylene peak at 3054 cm^-1^ disappeared and a carbonyl peak appeared at 1722 cm^-1^ indicating oxidization (Figure 2D). Collectively, these data show that Ce6 enhances ^1^O_2_ generation, which activates the co-localized MEHPPV into excited MEHPPV*, which subsequently relaxed from the excited singlet state (S_1_) to the ground state (S_0_), releasing photons as afterglow 39, 40. With the addition of Ce6, the fluorescence of MEHPPV at 595 nm in the FCMC system under excitation at 500 nm was significantly reduced, the fluorescence lifetime became longer, and a new fluorescence emission peak appeared at 680 nm (Figure S11). At the same time, compared with the afterglow emission spectrum of F127@MEHPPV, the afterglow emission spectrum of FCMC exhibited strong afterglow signals at both 580 nm and 680 nm (Figure S12). This spectral change confirmed the effective ET from MEHPPV to Ce6, as well as the possibility of near-infrared window imaging achieved through ET. Furthermore, the afterglow intensity and fluorescence intensity of FCMC, as well as the fluorescence of SOSG fluorescence, exhibited dependence on the concentration of FCMC (Figure 2E and Figure S13), which indicated that the amount of ^1^O_2_ is boosted by FCMC (Figure 2F). Moreover, the ^1^O_2_ yield and afterglow intensity were directly correlated (R^2^ = 0.9825) (Figure 2G).

### Ce6 enhanced Cu^2+^ releasing from CuET

**Figure 3:**
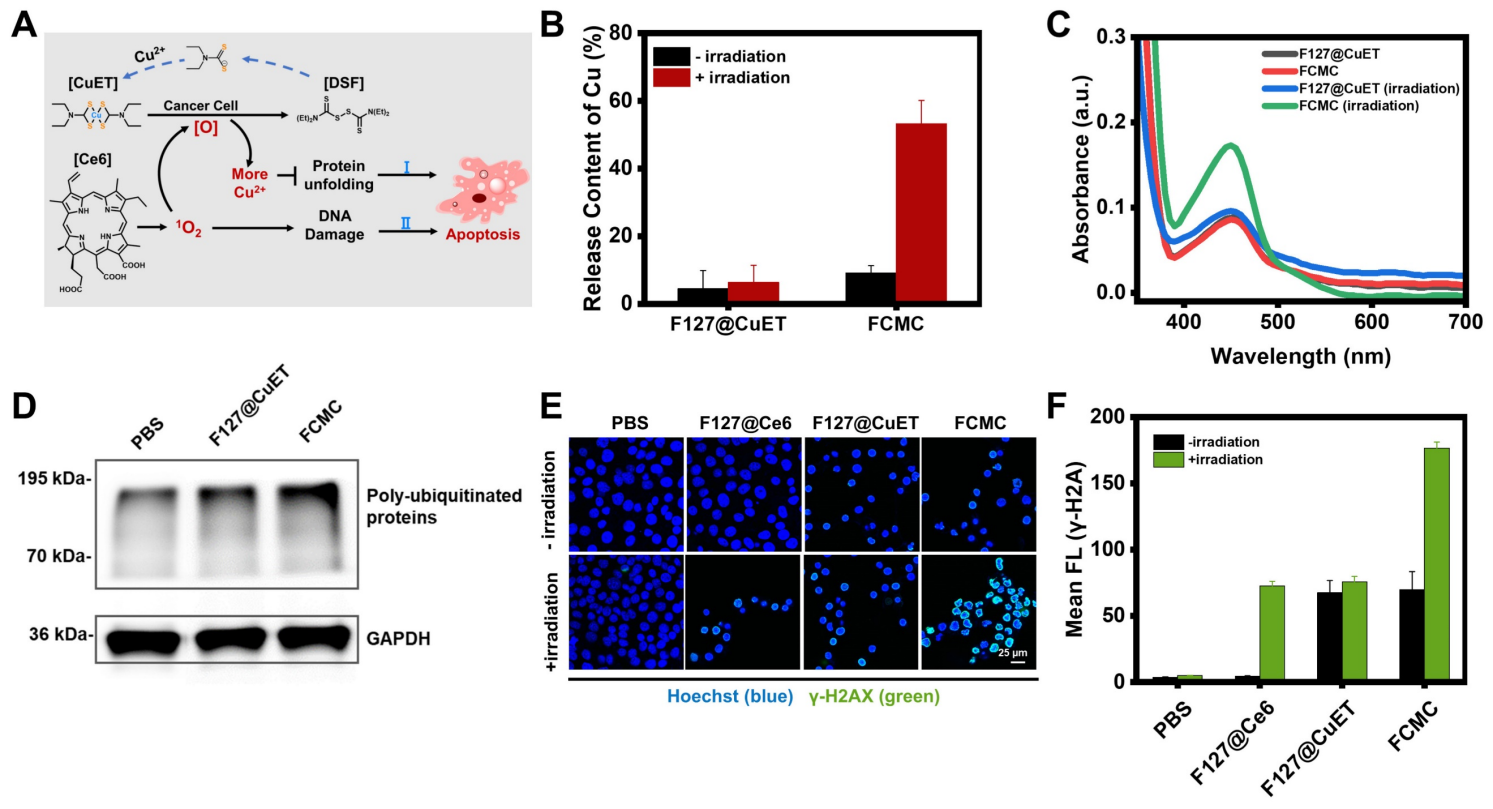
** Investigation the anti-tumor mechanism of FCMC.** (**A**) Schematic diagram of Ce6 enhancing Cu^2+^ release to improve therapeutic efficacy. ([O], ROS). (**B**) Efficiency of Cu^2+^ release from F127@CuET and FCMC under different conditions. (**C**) ^1^O_2_ triggered Cu^2+^ release from CuET in cells. (**D**) Western blot analysis of poly-ubiquitinated protein and GAPDH expression levels in 4T1 cells incubated under different conditions. (**E**) DNA damage of 4T1 cells treated with different conditions (green color indicates DNA damage). (**F**) Quantification of fluorescence intensities of each group in (**E**).

Previous reports indicated that CuET can bypass the copper transporter system and inhibit the function of p97 by releasing Cu^2+^ under oxidative conditions 41. Elevating the level of ROS in the tumor microenvironment has also been proven to be an effective strategy for accelerating the release of Cu^2+^ and the regeneration of CuET 42. The released Cu^2+^ disrupts the zinc finger motifs of Npl4, locking the essential conformational switch of the complex and inhibiting p97 unfolding, thereby causing apoptosis (Figure 3A). ICP-MS was then used to investigate whether Ce6 could increase Cu^2+^ release from CuET by analyzing the Cu^2+^ level under different conditions, including F127@CuET and FCMC, with or without irradiation. The results revealed a significantly higher Cu^2+^ content in the FCMC system than that of F127@CuET (Figure 3B), suggesting that ^1^O_2_ generated by Ce6 enhanced Cu^2+^ releasing from CuET under irradiation. Next, the potential of ^1^O_2_ to promote the release of Cu^2+^ at the cellular level was explored. Before that, cellular uptake behavior of FCMC was assessed by confocal laser scanning microscopy and flow cytometry analysis, and the results showed that the optimized incubation time for cell cellular uptake was 2 h (Figure S14). Then, the distribution of FCMC in cells using colocalization assay was investigated (Figure S15). According to the Pearson correlation coefficient, it could be inferred that FCMC was mainly distributed in the lysosome and mitochondrion. Next, the amount of Cu^2+^ was measured by using 2,9-dimethyl-1,10-phenanthroline (DMAB) spectrophotometry. The results showed that the absorbance of FCMC at 450 nm after irradiation was significantly higher than F127@CuET (Figure 3C). This result further proved that ^1^O_2_ could trigger Cu^2+^ releasing from FCMC. To further elucidate the mechanism of the antitumor activity of Ce6 and CuET in FCMC, an analysis of the expression of poly-ubiquitinated proteins in 4T1 cells after different treatments was conducted. As shown in Figure 3D, compared with the PBS group and the F127@CuET group, there was a significant accumulation of poly-ubiquitinated proteins in 4T1 cells treated with FCMC. These results indicate that FCMC could induce the production of ROS by introducing Ce6, which promotes the accumulation of ubiquitinated proteins, thereby achieving a more significant antitumor effect.

### DNA damage assay

Excess intracellular ROS cause lipid peroxidation, base oxidation, and double-stranded nucleic acid breaks (DSBs), resulting in spontaneous apoptosis 43. To confirm the ability of FCMC to cause DNA decomposition, gel electrophoresis of a random double-stranded DNA fragment (Table S2) treated with FCMC was performed. The results indicated that irradiation alone does not induce significant nucleic acid damage, whereas the addition of FCMC induces DNA breakage and the decrease of dsDNA correlated with increased irradiation time, consistent with increased ^1^O_2_ (Figure S16). In addition, DSBs are often associated with the phosphorylation of H2A histone family member X protein (H2AX) to form γ-H2AX 44. Therefore, DSBs induced by FCMC were further studied in cells using confocal microscopy. The results showed that FCMC effectively caused nucleic acid cleavage in 4T1 cells under irradiation, and resulted in increased γ-H2AX, as evidenced by immunostaining (Figure 3E and 3F).

### Antitumor and afterglow activities of FCMC *in vitro*

**Figure 4:**
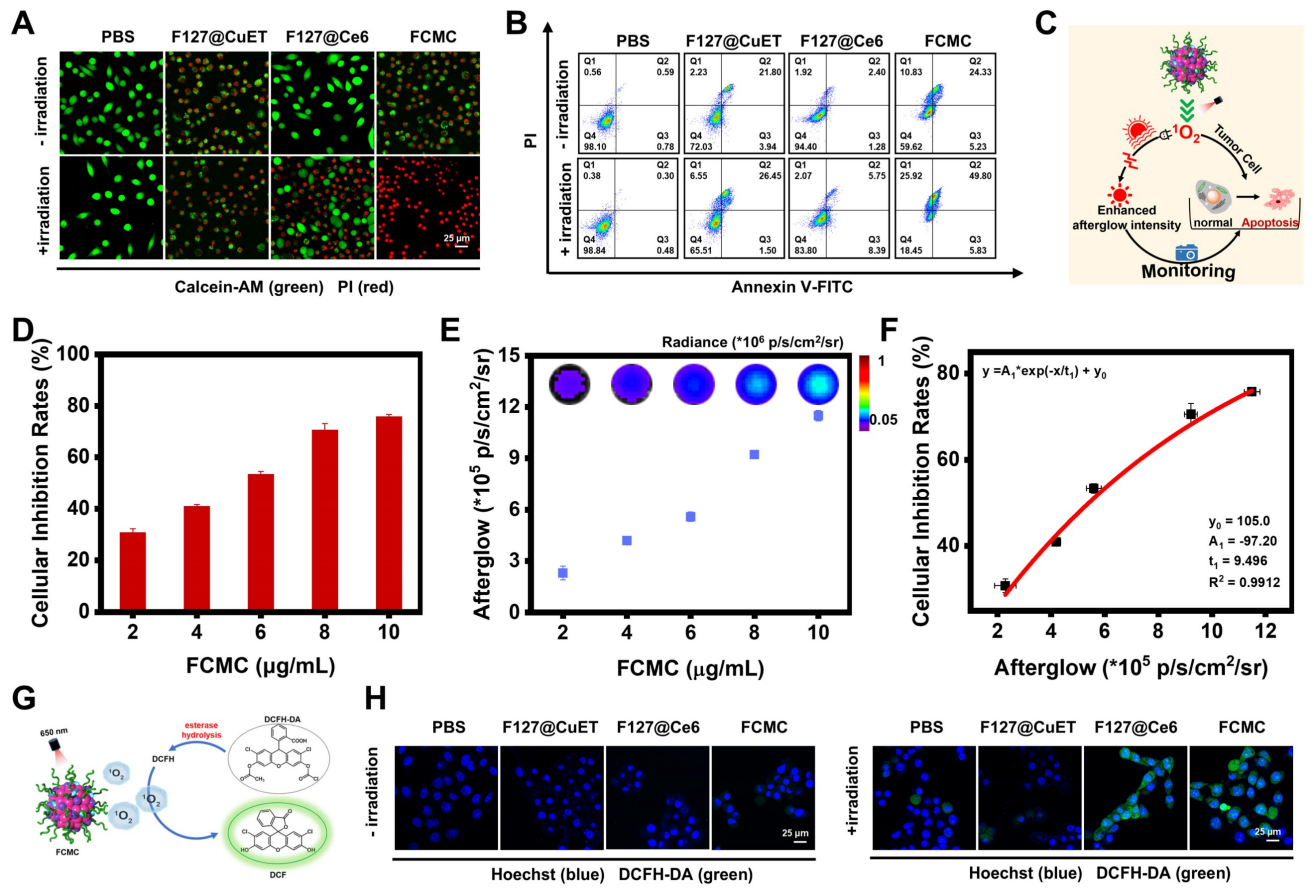
** Antitumor activity of FCMC *in vitro*.** (**A**) Fluorescent confocal images of live/dead cells after different treatments. Live and dead cells stained with calcination-AM (green) and PI (red), respectively. (**B**) Cell flow cytometry analysis of 4T1 cells treated with different conditions. (**C**) Schematic diagram showing the afterglow monitoring of ^1^O_2_ generation and correlation with FCMC-triggered cell death. (**D**) Cellular inhibition rates at different concentrations of FCMC. (**E**) Afterglow images and afterglow luminescence intensity at different concentrations of FCMC. (**F**) Correlation between afterglow intensity and cellular inhibition rates. (**G**) The detection mechanism of DCFH-DA for intracellular ROS. (**H**) Fluorescent confocal images of 4T1 cells incubated with different conditions. Cell nuclei and ^1^O_2_ were detected using Hoechst and DCFH-DA, respectively.

3-(4,5-dimethyl-2-thiazolyl)-2,5-diphenyl-2-H-tetrazolium bromide (MTT) cytotoxicity assays were performed to evaluate whether Ce6 and CuET can improve the therapeutic outcomes of DSF. As shown in Figure S17, CuET exhibited greater cytotoxicity toward 4T1 cells than free DSF, but much lower than FCMC (Figure S18A and S18B). These results were also validated in MCF-7 cells (Figure S18C and S18D). Calcination-AM and propidium iodide (PI) staining of 4T1 cells revealed that, compared with the control groups treated with PBS, F127@CuET, and F127@Ce6 with 650 nm laser irradiation, cells treated with FCMC exhibited greater fluorescent signals (red channel) indicative of dead cells (Figure 4A). In addition, the apoptosis rate of cancer cells was detected by flow cytometry using Annexin V-FITC and PI staining. Results from this analysis indicated that FCMC-treated 4T1 cells exhibit a higher apoptosis rate than cells in other control groups under identical laser irradiation conditions (Figure 4B), which signifies that FCMC induces significant cell inhibition.

The correlation between afterglow luminescence and ^1^O_2_ content prompted the applicability of afterglow for detecting anticancer activity *in vitro* (Figure 4C). The antitumor activity induced by FCMC was verified by incubating 4T1 cancer cells with different FCMC concentrations. Cell proliferation gradually decreased with increased FCMC concentration (Figure 4D), while the afterglow intensity and fluorescence intensity of treated cells also increased (Figure 4E and Figure S19). Importantly, a correlation was obtained between cancer cell inhibition rate and afterglow intensity (R^2^ = 0.9912) (Figure 4F). When cells were incubated with FCMC and ROS indicator, DCFH-DA, both Ce6- and FCMC-treated cells exhibited a DCFH-DA fluorescence signal, confirming ^1^O_2_ generation (Figure 4G and 4H).

### Antitumor and afterglow activities of FCMC *in vivo*

**Figure 5:**
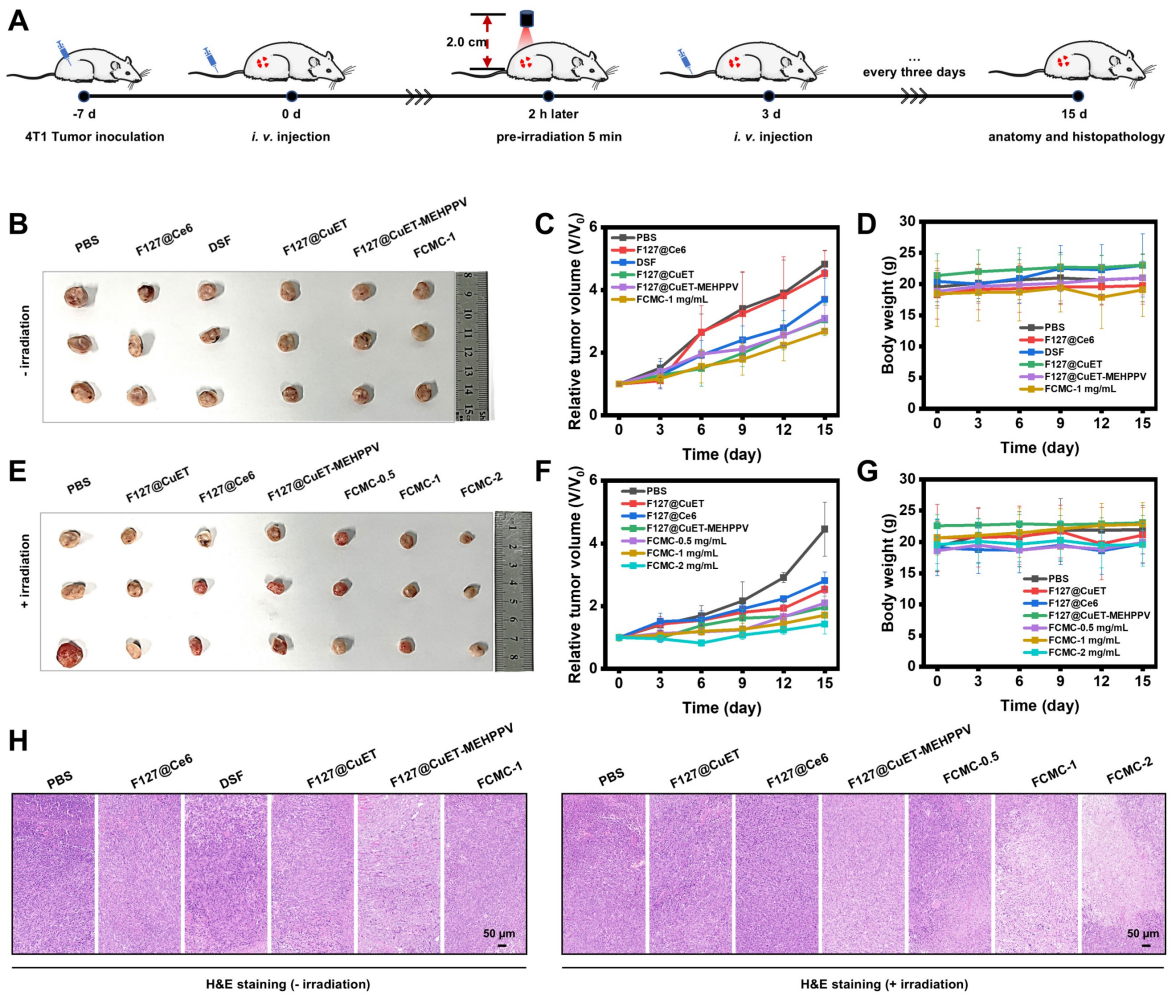
** Anticancer activity *in vivo*.** (**A**) Construction and treatment of BALB/c tumor-bearing 4T1 mouse model. (**B**) and (**E**) Digital photographs of excised tumors after 15 days of treatment (n=3). (**C**) and (**F**) Relative tumor volume for each group. (**D**) and (**G**) Body weight for each group 15 days after *i.v.* injection. (**H**) H&E staining of tumor slices after 15 days of treatment.

The tumor inhibition by FCMC was then carried out* in vivo* according to the procedure as shown in Figure 5A. Female BALB/C mice bearing 4T1 tumors were established by subcutaneous injection of 1×10^6^ 4T1 cells into the right dorsal region. The tumor-bearing mice subsequently received the following treatments every 3 days via intravenous (*i.v.*) injection: (1) PBS; (2) F127@Ce6; (3) DSF; (4) F127@CuET; (5) F127@CuET-MEHPPV; (6) FCMC-1 mg/mL; (7) PBS with irradiation; (8) F127@CuET with irradiation; (9) F127@Ce6 with irradiation; (10) F127@CuET-MEHPPV with irradiation; (11) FCMC-0.5 mg/mL with irradiation; (12) FCMC-1 mg/mL with irradiation; (13) FCMC-2 mg/mL with irradiation. The antitumor effect of FCMC was evaluated *in vivo* by monitoring tumor volume and body weight every 3 days. Analysis revealed that the tumor growth in the FCMC treatment group was significantly inhibited compared to the F127@CuET-MEHPPV group, with a tumor growth inhibition rate of 67.89% after 15 days of treatment. The above results indicated a synergistic antitumor effect between CuET and Ce6. (Figure 5B-G). Throughout the treatment, there was no significant change in body weight (Figure 5D and 5G), indicating a good safety profile. Tumors were collected after *i.v.* injection, and hematoxylin and eosin (H&E) staining revealed that the tumors treated with FCMC and irradiation exhibited apoptosis as evidenced by cell shrinkage and prominent necrosis with disappearing cell nuclei. This observation suggests an improved CuET chemotherapeutic activity (Figure 5H). Histological analysis showed no significant pathological changes in the heart, liver, spleen, lung, or kidney of mice treated with FCMC with irradiation compared with the PBS-treated group (Figures S20 and S21). The potential *in vivo* side effects of FCMC with irradiation was further evaluated by blood biochemical analyses. Results from this study indicated no significant difference in blood biochemical and hematological analysis parameters between the treatment and control groups (Figure S22). Therefore, FCMC can effectively inhibit tumor growth with negligible side effects at the experimental dosage.

To evaluate the applicability of FCMC as an afterglow imaging agent, the optical stability and tissue luminescence ability of FCMC were first investigated. The results showed that, even after 4 cycles irradiation of FCMC placed in black 96-well plates under a 650 nm laser, it still exhibited a higher afterglow signal than F127@MEHPPV (Figure S23). This observation may imply its low background noise. In addition, the ability of FCMC to generate afterglow luminescence can be retained by storing at -20°C after pre-irradiation. Afterglow signal intensity of FCMC can be recovered to 58% of the maximum value after 24 h of storage, indicating longevity imaging capability (Figure S24). Next, chicken tissues with different thickness (2 mm, 4 mm, 6 mm, and 8 mm) were placed on top of FCMC and the afterglow signals were recorded. As shown in Figure S25A-C, even though the afterglow signals decreased with increased chicken tissue thickness, an afterglow signal remained detectable even at a thickness of 8 mm. The fluorescence signals passing through the chicken tissue at different depths were simultaneously recorded, and comparison of the SBR indicated that after covering with 2 mm chicken tissue, the SBR of afterglow was 16.47 times higher than that of fluorescence (Figure S25D and S25E). This suggested that the afterglow luminescence performance of FCMC was more beneficial for imaging *in vivo*.

**Figure 6:**
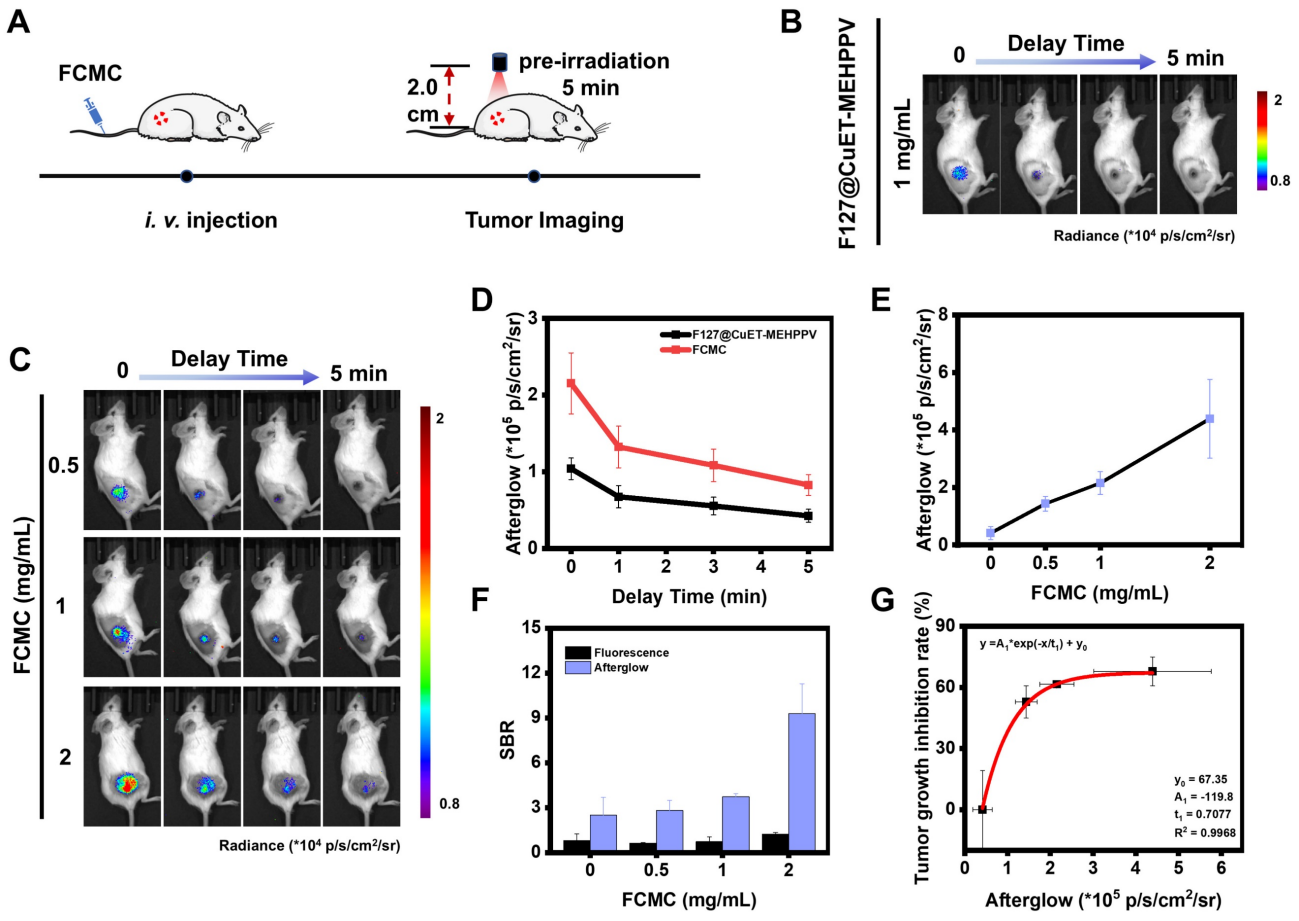
**Correlation between afterglow luminescence and antitumor efficiency *in vivo*.** (**A**) Schematic diagram of afterglow luminescence imaging of mice bearing subcutaneous 4T1 xenograft tumors*.* (**B**) Afterglow images of mice bearing subcutaneous 4T1 xenograft tumors *i.v.* injected with F127@CuET-MEHPPV (1 mg/mL). (**C**) Afterglow images of mice bearing subcutaneous 4T1 xenograft tumors *i.v.* injected with FCMC (0.5, 1, and 2 mg/mL) (**D**) Statistics of afterglow signal intensity of the tumor site after *i.v.* injection of F127@CuET-MEHPPV (1 mg/mL) and FCMC (1 mg/mL). (**E**) Statistics of afterglow signal intensity of the tumor site after *i.v.* injection of FCMC (0.5, 1, and 2 mg/mL). (**F**) The SBR for afterglow and fluorescence images. (**G**) Correlation between afterglow luminescence imaging and tumor inhibition rates *in vivo*.

Before *in vivo* afterglow imaging, we first assessed the ability of FCMC to target the tumor area using a tumor-bearing mouse model. FCMC was *i.v.* injected into the tumor-bearing mice via the tail vein, and the fluorescence intensity in the tumor area was monitored at different time intervals. Due to the enhanced permeability and retention (EPR) effect, the fluorescence signal at the tumor site gradually increased and reached a maximum value within 1-2 h, after which the signal in that area gradually weakened (Figure S26). This indicated that FCMC could rely on the EPR effect to demonstrate rapid and efficient tumor targeting capabilities when administered via *i.v.* injection at 1 h. Next, 4T1 tumor-bearing mice were *i.v.* injected with F127@CuET-MEHPPV or FCMC, and afterglow/fluorescence image data were collected and analyzed (Figure 6A). In this experiment, the afterglow signal was immediately collected once the light irradiation stopped so as to minimize the interference caused by different time points as much as possible. The tumor area exhibited strong afterglow contrast, while no obvious signals were detected elsewhere (Figure 6B and 6C). However, the fluorescence images exhibited a relatively strong background fluorescence (Figure S27A). In addition, compared with the group injected with F127@CuET-MEHPPV, mice receiving FCMC exhibited greater afterglow intensity. Moreover, FCMC exhibited a stronger and longer afterglow signal decay time than F127@CuET-MEHPPV (Figure 6D). These results verify that Ce6 doping can improve *in vivo* afterglow imaging efficiency. At the same time, the fluorescence and afterglow signals at the tumor site after *i.v.* injection of FCMC at different concentrations were also monitored. The results showed that with the increase in the concentration of *i.v.* injected FCMC, both the afterglow signal and the fluorescence signal in the tumor area gradually strengthened (Figure 6E and Figures S27B and S27C), and the SBR in afterglow imaging was higher than that in fluorescence imaging (Figure 6F). Furthermore, a correlation was obtained between the afterglow intensity of the tumor area and the tumor inhibition rate on day 15 of treatment (Figure 6G).

## Discussion

DSF has been identified as a potential chemotherapeutic agent for breast cancer treatment. As the anticancer activity of DSF is closely linked to the presence of Cu^2+^, DSF is typically administered concurrently with Cu^2+^-containing compounds, such as copper chloride. However, bioavailable copper is often bound to proteins and other ligands, and excessive free copper can lead to significant physiological toxicity. Furthermore, DSF is unstable during delivery and many reported nanocarriers have poor drug loading capacities. To address these issues, it is essential to increase the stability of DSF and reduce the dose of Cu^2+^ *in vivo* to minimize the risk of off-target effects and damage to normal tissues.

The approach in this study differs from other drug self-delivery systems in that it uses a self-boosting strategy for ^1^O_2_ production through Ce6 doping to enhance therapy efficacy. Our highly efficient FCMC nanoplatform is created through nanoprecipitation of hydrophobic interactions, and increases ^1^O_2_ generation and faster Cu^2+^ release from CuET. The combination of augmented CuET chemotoxicity and Ce6 photodynamic toxicity creates a synergistic effect, thereby achieving significant therapeutic benefit.

Intelligent nanoplatforms for imaging-guided therapy have gained increasing attention, with afterglow imaging offering higher SBR and deeper tissue penetration than fluorescence imaging. In our FCMC system, the ^1^O_2_ generated from Ce6 not only facilitates tumor cell killing, but also acts as a nucleophilic species that activates MEHPPV to high-energy intermediates that enhance afterglow intensity. We established a correlation between afterglow intensity and ^1^O_2_ yield in solution. Moreover, we demonstrated a relationship between afterglow intensity and tumor inhibition *in vitro* and *in vivo*. The afterglow intensity may provide access to previously unknown treatment information that can be used for predicting therapeutic outcomes and for dynamically adjusting treatment parameters, including dosage and irradiation time.

## Conclusion

In summary, we have developed a novel drug self-delivery system by repurposing DSF for efficient cancer therapy. By enhancing the amount of ^1^O_2_ in the tumor and Ce6-enhanced Cu^2+^ release, FCMC induced significant apoptosis and antitumor activity. Furthermore, the afterglow properties of FCMC allowed for high-contrast afterglow imaging. Our study provides an efficient approach for enhancing the efficacy of approved drugs in tumor treatment while minimizing toxic side effects. This low-cost, reliable strategy may be generalized to extend the potential application of existing drugs.

## Supplementary Material

Supplementary materials and methods, figures.
