# Supplementary material for: Dual-purposing disulfiram for enhanced chemotherapy and afterglow imaging using chlorin e6 and semiconducting polymer combined strategy
Source: Theranostics. 2024 Aug 19;14(13):5141–51. doi: 10.7150/thno.96136 (PMC11388071; doi:10.7150/thno.96136)
Supplement: Supplementary file 1 — Supplementary materials and methods, figures. [file thnov14p5141s1.pdf]

Supplementary materials

Dual-purposing disulfiram for enhanced chemotherapy and afterglow imaging using chlorin e6 and semiconducting polymer combined strategy

Di Zhao [1], Aifang Zhou [1], Xintong Dong [1], Hong-Min Meng [1] ✉, Yating He [1], Lingbo Qu [1], Ke Zhang [2], Yuehe Lin [3], Zhaohui Li [1] ✉

1. College of Chemistry, Henan Joint International Research Laboratory of Green Construction of Functional Molecules and Their Bioanalytical Applications, Zhengzhou University, Zhengzhou 450001, P. R. China
2. Department of Chemistry and Chemical Biology, Northeastern University, Boston, Massachusetts 02115, United States
3. Department of Chemistry, School of Mechanical and Materials Engineering, Washington State University, Pullman, Washington 99164, United States

✉ Corresponding authors: Email: hmmeng2017@zzu.edu.cn (H. Meng), zhaohui.li@zzu.edu.cn (Z. Li).

E-mail addresses: hmmeng2017@zzu.edu.cn (H. Meng), zhaohui.li@zzu.edu.cn (Z. Li).

Experimental Section

Materials.

Disulfiram was purchased from J&K Scientific Co., Ltd. (Beijing, China). Poly[2-methoxy-5-(2-ethylhexyloxy)-1,4-phenylenevinylene] (MEHPPV), chlorin e6 (Ce6), 2′,7′-dichlorodihydrofluorescein diacetate (DCFH-DA), and pluronic-F127 (F127) were purchased from Sigma-Aldrich. Hoechst 33342, LysoTracker™ Deep Red, MitoTracker™ Deep Red FM, and Calcein green AM (Calcein-AM) were purchased from Thermo Fisher Scientific. DNA damage assay kit and singlet oxygen sensor green (SOSG) were purchased from Beyotime Biotechnology. Ubiquitin (P37) antibody was purchased from Cell Signaling Technology, Inc. Goat Anti-Rabbit IgG H&L (HRP) was purchased from Zen-Bioscience Co., Ltd. Recombinant Anti- GAPDH antibody (Rabbit mAb) was purchased from Wuhan Servicebio Technology Co., Ltd. 2,9-Dimethyl-1,10-phenanthroline (DMAB) was purchased from Alfa Chemical Co., Ltd. Annexin V-FITC/PI apoptosis detection kit and thiazolyl blue tetrazolium bromide (MTT) were purchased from Sangon Biotech Co., Ltd (Shanghai, China). Hydroxylamine hydrochloride and 2,2,6,6, -tetramethyl-4-piperidone (TEMP) was purchased from Sun Chemical Technology Co., Ltd (Shanghai, China). The chicken tissues were purchased from local market. The female BALB/c mice were purchased from SPF Biotechnology Co., Ltd. (Beijing, China).

Apparatus.

Transmission electron microscope (TEM) images were obtained on JEM-2100 microscope (JEOL, Japan). Dynamic light scattering (DLS) and zeta potential were performed on Zetasizer Nano ZS90 (Malvern, England). UV-Vis spectra were recorded on U-3900H spectrophotometer (Hitachi, Japan). Fluorescence spectra were carried out on F-7100 fluorescence spectrometer (Hitachi, Japan). Fourier transform infrared spectra was performed on Tensor 27 spectrometer (Bruker, Germany). X-ray photoelectron spectroscopy (XPS) was recorded through ESCALab250 (Thermo Scientific, USA). Cells images were collected by a Leica TCS SP8 confocal laser scanning fluorescence microscope (Leica, Germany). MTT assay was measured by a Spark® Multimode Microplate Reader (Tecan, Switzerland). Flow cytometry analysis was obtained *via* Flow Cytometer (BD, USA). The Cu^{2+} was analyzed by 8900 Triple Quadrupole ICP-MS (Agilent, USA). $^{1}O_2$ production was confirmed by EMX-12 electron paramagnetic

resonance (EPR) spectrometer (Bruker, Germany). Afterglow luminescence images were obtained on IVIS® Spectrum *in vivo* imaging system (PerkinElmer, USA).

Preparation of CuET.

CuET was prepared *via* a coordination self-assembly strategy. Typically, 0.3 mmol $CuSO_4•5H_2O$ and 0.1 mmol DSF were first dissolved in 5 mL N, N-dimethylformamide (DMF), respectively and then $CuSO_4$ solution was added into DSF solution drop by drop. The mixed solution was stirred for 1 h at room temperature to obtain brownish green precipitate. The precipitate was collected by centrifugation (5000 rpm, 15 min) and freeze-dried for further use.

Preparation of Various Semiconducting Polymer Nanoparticles.

For synthesis of F127@CuET, a mixed THF solution (1 mL) containing CuET (0.4 mg) and F127 (4 mg) was rapidly injected into 9 mL deionized water under sonication for 10 min. THF was then removed by rotary evaporator. For synthesis of F127@Ce6, a THF mixture solution (1 mL) containing Ce6 (0.4 mg) and F127 (4 mg) was rapidly injected into 9 mL water under continuous sonication. For synthesis of F127@CuET-MEHPPV, a THF mixture solution (1 mL) containing MEHPPV (0.4 mg), CuET (0.4 mg) and F127 (4 mg) was rapidly injected into 9 mL water under continuous sonication.

For preparation of F127@CuET-MEHPPV-Ce6 (FCMC), a THF mixture solution (1 mL) containing MEHPPV (0.4 mg), Ce6 (0.4 mg), CuET (0.4 mg) and F127 (4 mg) was rapidly injected into 9 mL water under continuous sonication. THF was evaporated after 10 min of sonication. Water was chosen to wash the resulted nanoparticles in a 100 KD centrifugal filter tube for three times to purify the product. The final obtained precipitate was dispersed in PBS.

To determine the doping ratio in these samples, the absorbance intensities of each component in the supernatant were measured using the UV standard curve formulas of CuET, MEHPPV, and Ce6 to calculate the loading amount.

Stability Assay of FCMC.

The stability of FCMC was investigated by recording the released DSF and Cu^{2+} from FCMC under different conditions. For measuring DSF, FCMC was incubated in PBS with different pH for varied periods, and then the UV-Vis spectra of collected supernatant was recorded. For measuring Cu^{2+}, the collected supernatant was first dialyzed through a 0.22 μm filter membrane

and then quantified by ICP-MS. In addition, the stability of the FCMC was also assessed *via* monitored the size change by DLS.

Singlet Oxygen Detection in Solution.

Singlet oxygen sensor green (SOSG) was used to evaluate the singlet oxygen production by FCMC in solution. Different concentrations of FCMC were mixed with 1 μM SOSG and irradiated by 650 nm laser. The generation of $^1O_2$ was then evaluated by measuring the fluorescence intensity of SOSG at 540 nm.

$^1O_2$ Triggered Cu^{2+} Release Assay.

Four experimental groups were designed, namely F127@CuET group (50 μg/mL) with or without irradiation, FCMC group (50 μg/mL) with or without irradiation.

In solution assay: The above-mentioned groups were treated by illumination or without illumination, and further were incubated for 4 h. Then, the supernatant solution of each group was collected by centrifugation. The content of copper ions was measured using ICP-MS after fully digesting and diluting the solution with aqua regia.

In cells assay: Firstly, 4T1 cells were seeded into a 6-well plate. 4T1 cells were treated by F127@CuET or FCMC (50 μg/mL) at 37 °C and 5% CO_2 for 2 h, followed by exposure to light for 5 min or kept in the dark. They were then continued to be incubated in the incubator for another 2 h. Then, cells were collected using trypsin-EDTA solution, and washed 3 times with metal-free DPBS. Next, 400 μL lysis buffer was added to each group of cell pellets. The cell suspensions were collected to measure the protein content. Finally, the cell suspension was digested with concentrated hydrochloric acid and 30% H_2O_2, and the copper content was analyzed using 2,9-dimethyl-1,10-phenanthroline probe in the presence of hydroxylamine hydrochloride.

Confocal Fluorescence Imaging of FCMC.

Cellular uptake behavior of FCMC: 4T1 cells were seeded in confocal dishes (1×10^5/dish) for 24 h, and then treated with FCMC (20 μg/mL) for 0, 1, 2, 3, and 4 h, respectively. Finally, the cells were incubated with Hoechst 33342 for 15 min and subjected to imaging.

For the colocalization assay, 4T1 cells were first incubated with FCMC (20 μg/mL) for 2 h and then incubated with the commercial Hoechst 33342, LysoTracker™ Deep Red, and

MitoTracker™ Deep Red FM for 15 min. The Pearson correlation coefficient was determined by Image J software.

Cell Culture and Intracellular Singlet Oxygen Imaging.

4T1 cells and MCF-7 cells were cultured in DMEM (Dulbecco's Modified Eagle Medium) supplemented with 10% FBS and 100 μg/mL penicillin-streptomycin. The cells were maintained in an atmosphere of 5% CO_2 at 37 °C. For imaging of $^1O_2$ generation, 4T1 cells were co-cultured with PBS, F127@CuET (20 μg/mL), F127@Ce6 (20 μg/mL), or FCMC (20 μg/mL) for 2 h and then treated with 650 nm laser irradiation for 5 min (300 mW/cm^2) or not. Then, those cells were stained with Hoechst (1 μg/mL) and DCFH-DA (15 μM) for 30 min, respectively. Finally, confocal laser scanning microscopy was used to image these cells. (Hoechst: λex = 405 nm; DCFH-DA: λex = 488 nm).

FCMC-Induced Nucleic Acid Break Assay.

To investigate the ability of FCMC induced nucleic acid damage, polyacrylamide gel electrophoresis (PAGE) was carried out. Simply put, a random double strand DNA (dsDNA) (detailed information for DNA sequence was listed in Table S2) was treated under different conditions: (1) 1 μM dsDNA; (2) 1 μM dsDNA with 650 nm laser irradiation for 5 min; (3) 1 μM dsDNA + 50 μg/mL FCMC; (4) 1 μM dsDNA +50 μg/mL FCMC with 650 nm laser irradiation for 5 min. Then, these samples were mixed with 6 × loading buffer and then loaded into 12% PAGE. The PAGE was implemented at 200 V for 10 min for pre-enrichment, and then changed to 80 V for 20 min. Finally, the gel was imaged on Bio-Rad Gel Doc™ EZ imager.

For γ-H2AX analysis, 4T1 cells were treated with PBS, F127@CuET (10 μg/mL), F127@Ce6 (10 μg/mL) or FCMC (10 μg/mL), respectively. After incubation for 2 h, those cells were irradiated with 650 nm laser (300 mW/cm^2, 5 min) or not. After incubation for another 2 h, those cells were stained with anti-phospho-histone γ-H2AX rabbit monoclonal antibody and further stained with Alexa Fluor 488-conjugated goat anti-rabbit secondary antibody. Cell nucleus was stained by Hoechst. Finally, confocal laser scanning microscopy was used to image these cells. (Hoechst: λex = 405 nm; Alexa Fluor 488: λex = 495 nm).

Western Blot Assay.

Western blot assay was used to detect the expression of ubiquitylated proteins. First, 4T1 cells were treated with PBS, F127@CuET, and FCMC (20 μg/mL) for 6 h, respectively. Then, the cells were pre-irradiated with 650 nm laser for 5 min and further cultured for another 6 h. Finally, the cells were collected by centrifugation (1500 rpm, 4 min). An appropriate volume of cell lysis buffer was added to the collected cells and lysed on ice for 30 min. Subsequently, the protein in the supernatant was collected by centrifugation at 12000 rpm, 4°C for 10 min, and the protein concentration was quantified using a BCA protein quantitative detection kit (Wuhan Servicebio Technology Co., Ltd.). The obtained protein samples were separated by sodium dodecyl sulfate-polyacrylamide gel electrophoresis (SDS-PAGE) and transferred to a polyvinylidene fluoride (PVDF) membrane. After blocking with 5% skim milk at room temperature for 30 min, the PVDF membrane was incubated with Ubiquitin (P37) antibody or the internal reference protein GADPH on a decolorizing shaker at 4°C overnight. After incubation, the PVDF membrane was washed three times with TBST buffer for 5 min, and then incubated with HRP-conjugated secondary antibody at room temperature for 30 min. Finally, the PVDF membrane was observed using a chemiluminescence imaging system (SCG-W3000, Wuhan Servicebio Technology Co., Ltd.).

Cytotoxicity Assay.

To study the cytotoxicity of different nanoparticles, 4T1 and MCF-7 cells were both cultured in 96-well plates for 24 h incubation. Then, these cells were separately incubated with various concentrations of DSF, F127@CuET, F127@Ce6, F127@CuET-MEHPPV, and FCMC for 6 h. These cells were then illuminated with 650 nm laser (300 mW/cm^2, 5 min) or not and further incubated for 18 h. Finally, the relative cellular viability was tested by 3-(4, 5-dimethylthiazol-2-yl)-2, 5-diphenyltetrazolium bromide (MTT) assay.

Cell Apoptosis and Live/Dead Cell Imaging Assay.

For cell apoptosis assay, 4T1 cells were incubated with PBS, F127@CuET (10 μg/mL), F127@Ce6 (10 μg/mL) or FCMC (10 μg/mL) for 24 h, and then treated with 650 nm irradiation (300 mW/cm^2, 5 min) or not. Subsequently, these cells were rinsed three times with PBS and then digested by trypsin. The collected cells were incubated in 1× binding buffer (200 μL)

containing Annexin V-FITC (5 μL) and PI (10 μL) for 15 min in the dark environment. Finally, apoptosis assay was analyzed by flow cytometry.

For live/dead cell imaging, calcination-AM (1 μg/mL) and PI (10 μL) were incubated with different nanoparticles (10 μg/mL) treated cancer cells to stain the live and dead cells, respectively. After incubation for 30 min, the cells were washed with PBS for several times and then imaged by confocal laser scanning microscope (calcination-AM: λex = 405 nm; PI: λex = 488 nm).

Tissue Penetration Depth Study.

FCMC (100 µg/mL) was first placed in a 96 well-plate and pre-irradiated with a 650 nm laser for 5 min (300 mW/cm^2). Chicken tissues with different thicknesses (2, 4, 6, and 8 mm) were then spread on the surface of the well plate. The afterglow luminescence images of chicken tissues were performed using 60 s of acquisition with the IVIS Spectrum imaging system. Fluorescence images were captured with 2 s acquisition time with excitation at 500 nm and emission at 680 nm using the IVIS Spectrum imaging system.

Tumor Mouse Model and Anticancer Efficacy *in vivo*.

6-8 weeks old female athymic BALB/c mice were inoculated subcutaneously with 100 μL of PBS containing 4T1 cells (1×10^6) to construct tumor-bearing mice model. All animal experiments conformed to the animal use and care regulations of local institutions and were approved by the Laboratory Animal Center of Henan Province.

For studying the anticancer efficacy of FCMC *in vivo*, mice bearing tumors were received the following treatment every 3 days *via i.v.* injection (n = 3): (1) PBS; (2) F127@Ce6; (3) DSF; (4) F127@CuET; (5) F127@CuET-MEHPPV; (6) FCMC (1 mg/mL); (7) PBS with irradiation; (8) F127@CuET with irradiation; (9) F127@Ce6 with irradiation; (10) F127@CuET-MEHPPV with irradiation; (11) FCMC (0.5 mg/mL) with irradiation; (12) FCMC (1 mg/mL) with irradiation; (13) FCMC (2 mg/mL) with irradiation. The tumor sizes and mouse body weights were continuously recorded for 15 days. Finally, blood biochemical analysis and H&E staining for the tumors and the main organs (heart, liver, kidney, lung, and spleen) from each group were performed. The tumor volume = $(\text{width}^2 \times \text{length})/2$.

***In vivo* Tumor Imaging.**

The constructed 4T1 tumor-bearing mice (n=3) were *i.v* injected with F127@CuET-MEHPPV (1.0 mg/mL) or FCMC (e.g., 0.5, 1, and 2 mg/mL) when tumors grown around 100 mm^3 in volume, respectively. Afterglow and fluorescence images were captured at different points after 650 nm laser irradiation (300 mW/cm^2, 5 min) of tumor site in mice. The afterglow luminous images were collected in the open filter mode through the IVIS Spectrum imaging system (60 s acquisition time). Fluorescence images were captured with 2 s acquisition time with excitation at 500 nm and emission at 600 nm or 680 nm using the IVIS Spectrum imaging system.

Supplementary tables

Table S1. The summary of the ratio of added precursors and the percentage of each component obtained for various FCMC nanocomposites.

Mass ratio of added precursors (CuET: MEHPPV: Ce6)	load efficiency (%)		
	CuET	MEHPPV	Ce6
1:1:0	28.87±0.44	85.13±0.09	
1:1:0.5	32.51±0.17	85.87±0.04	76.68±0.70
1:1:1	41.25±0.84	86.06±0.02	54.73±0.63
1:1:2	11.19±1.68	88.77±0.06	86.36±0.07
1:1:3	38.34±0.84	88.11±0.02	87.91±0.02

Table S2. Sequences of DNA used in this work.

Name	Sequence (5′-3′)
DNA-21	TAG CTT ATC AGA CTG ATG TTG A
Anti-DNA-21	TCA ACA TCA GTC TGA TAA GCT A

Supplementary figures

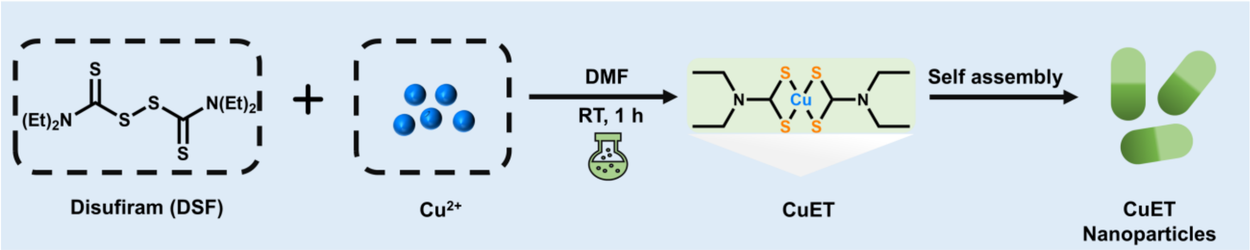

Figure S1. Schematic illustration of the preparation of CuET nanoparticles through self-assembly.

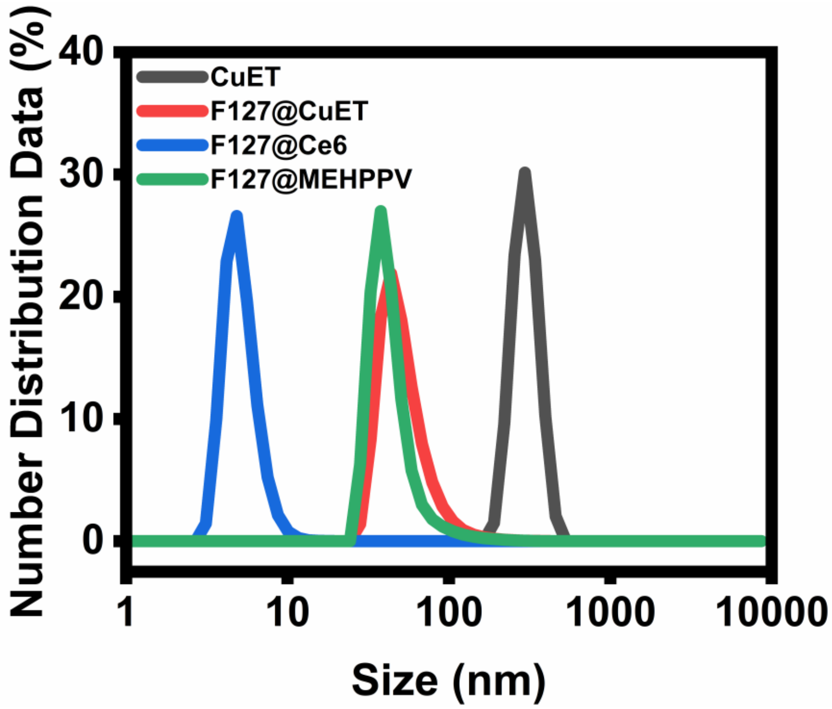

Figure S2. The hydrodynamic sizes of various nanoparticles.

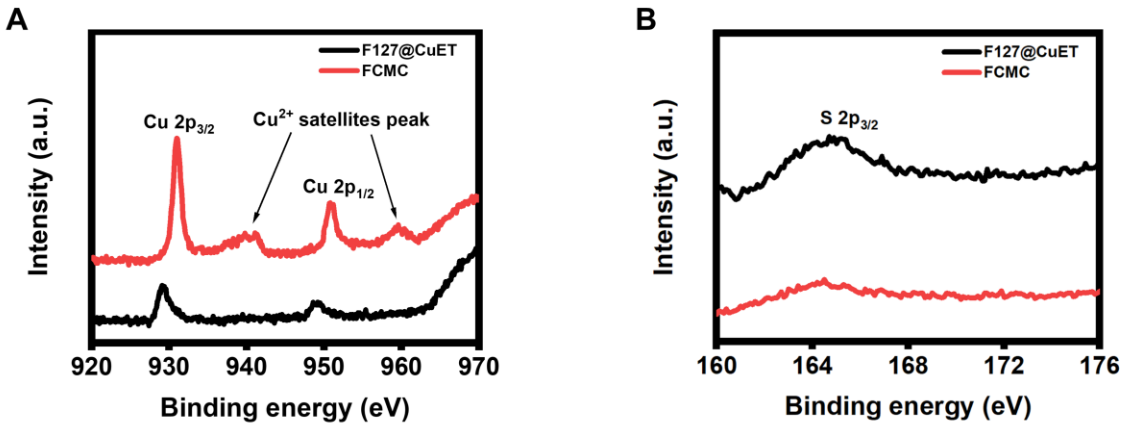

Figure S3. (**A**) Cu 2p XPS spectra of F127@CuET and FCMC. (**B**) S 2p XPS spectra of F127@CuET and FCMC.

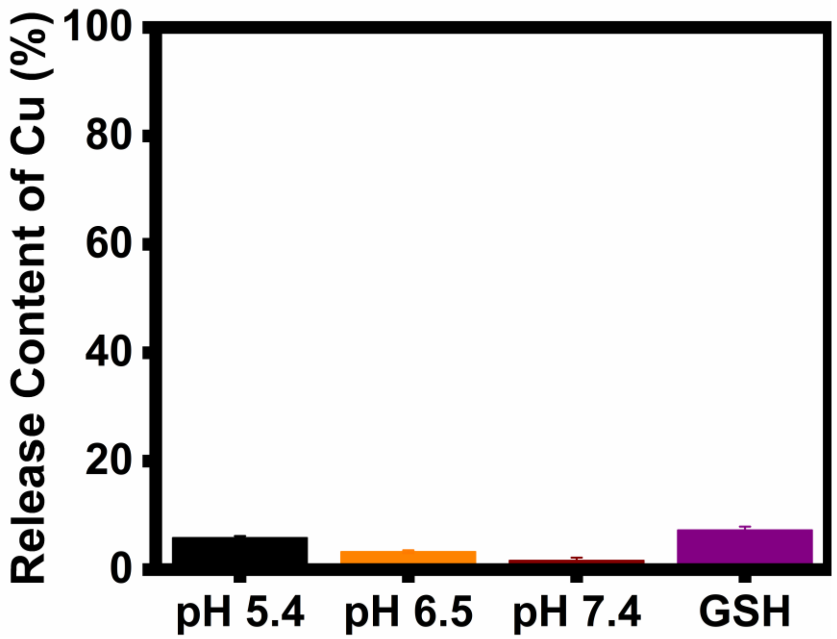

Figure S4. Release content of Cu in different PBS buffers (pH 5.4, pH 6.5, pH 7.4 and 1 mM GSH).

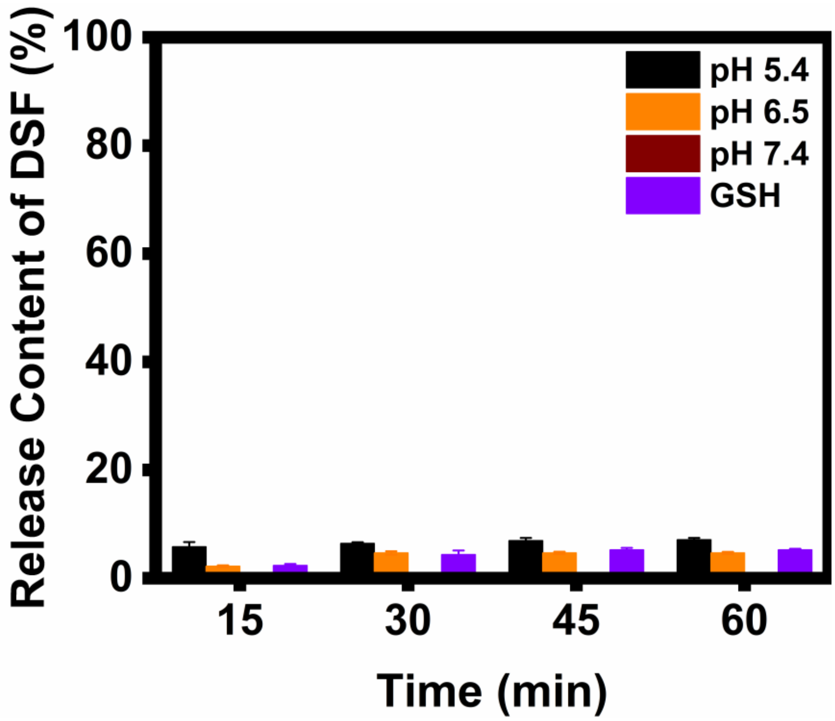

Figure S5. Release content of DSF at different time points in different PBS buffers (pH 5.4, pH 6.5, pH 7.4 and 1 mM GSH).

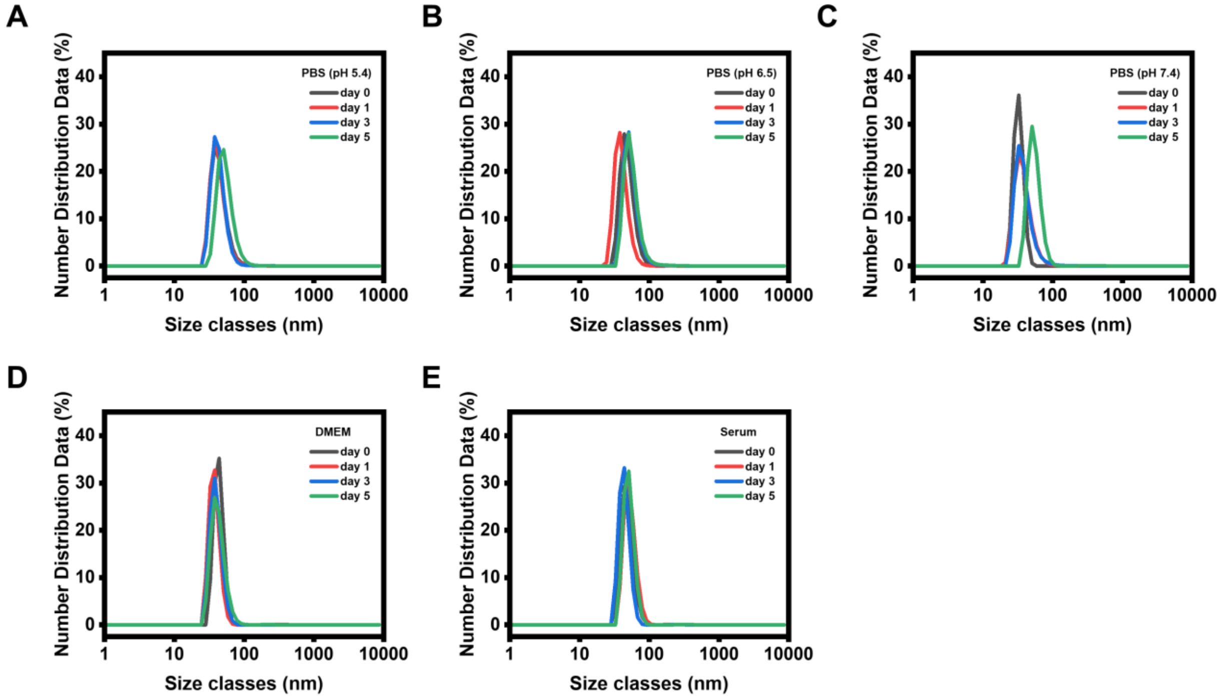

Figure S6. DLS sizes of FCMC in different solutions incubated for 5 days.

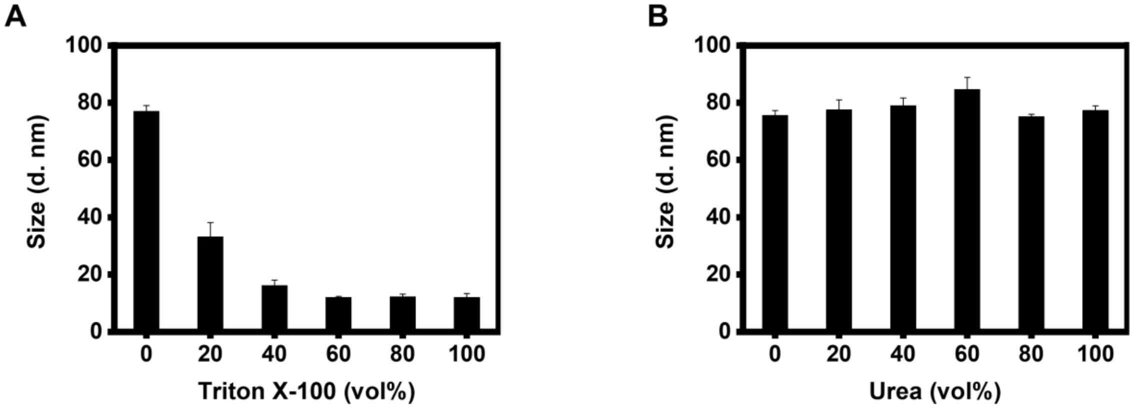

Figure S7. DLS sizes of FCMC in solutions containing different ratios of Triton X-100 (**A**) and urea (**B**).

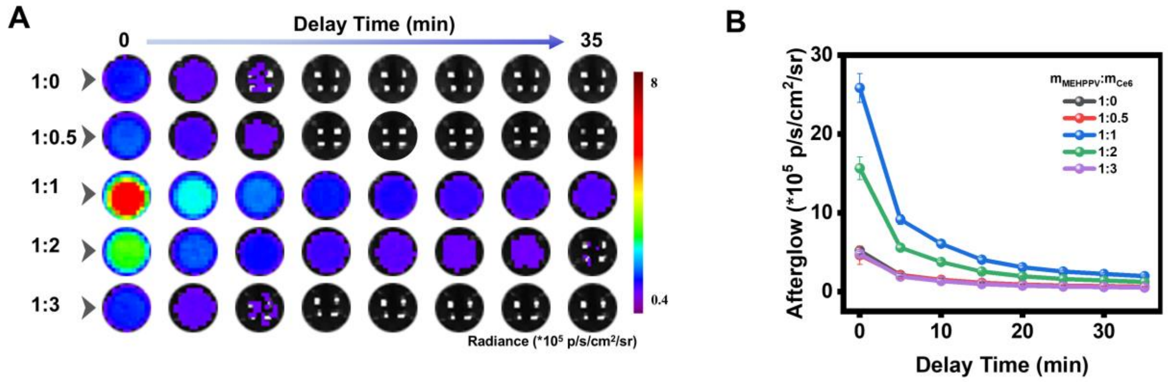

Figure S8. (**A**) The afterglow images and (**B**) afterglow signal statistics of MEHPPV/Ce6 with different doping ratios within 35 min after 5 min of illumination treatment.

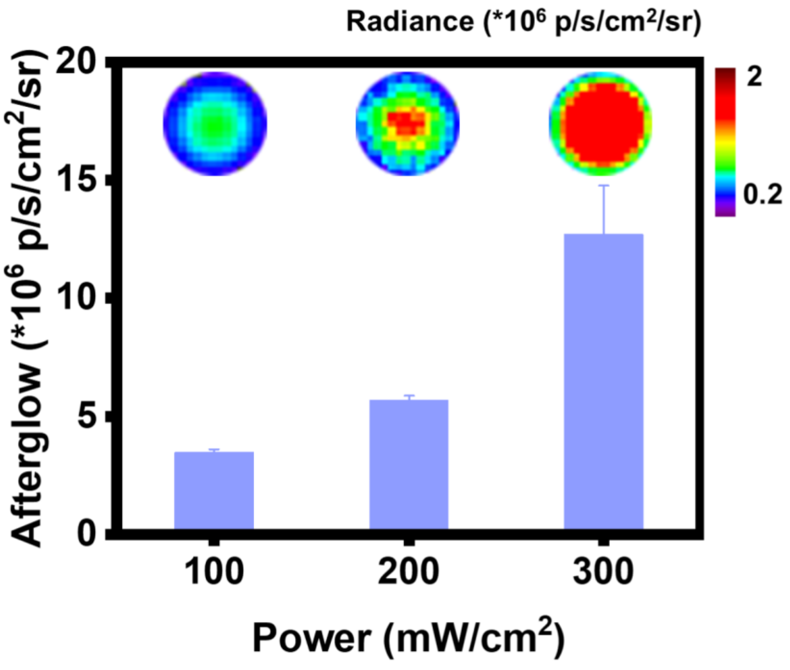

Figure S9. Afterglow images and afterglow intensity of FCMC after irradiation with different power levels.

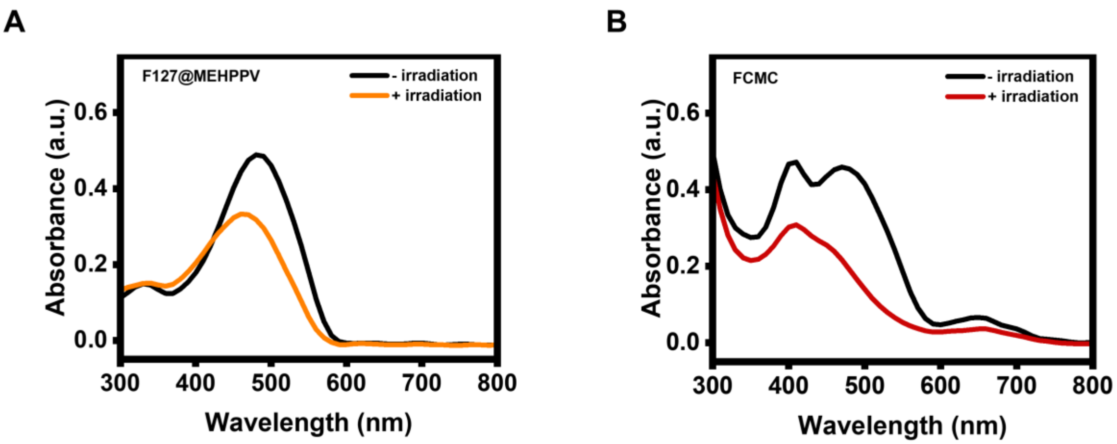

Figure S10. UV-Vis absorption spectra of (**A**) F127@MEHPPV and (**B**) FCMC before or after laser irradiation for 5 min.

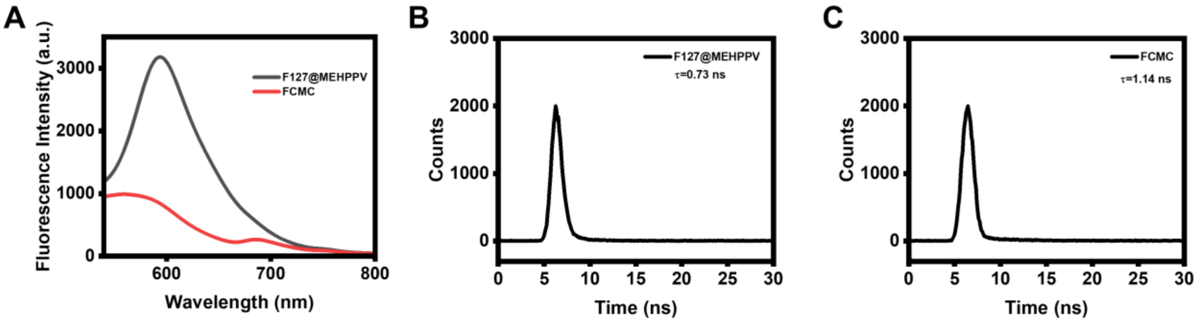

Figure S11. (**A**) Florescence emission spectra of F127@MEHPPV and FCMC. (**B**) Decay spectrum of F127@MEHPPV and FCMC with λex = 500 nm and λem = 595 nm.

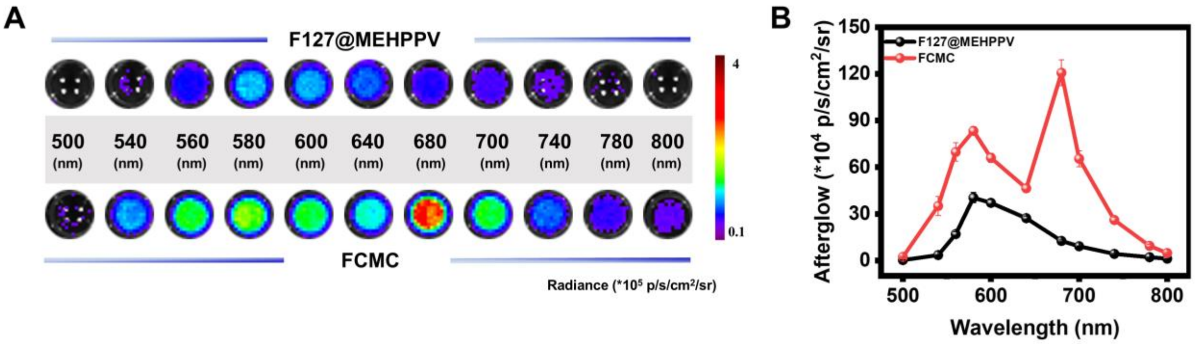

Figure S12. (**A**) Afterglow images of F127@MEHPPV and FCMC in different channels. (**B**) Afterglow luminescence spectra of F127@MEHPPV and FCMC.

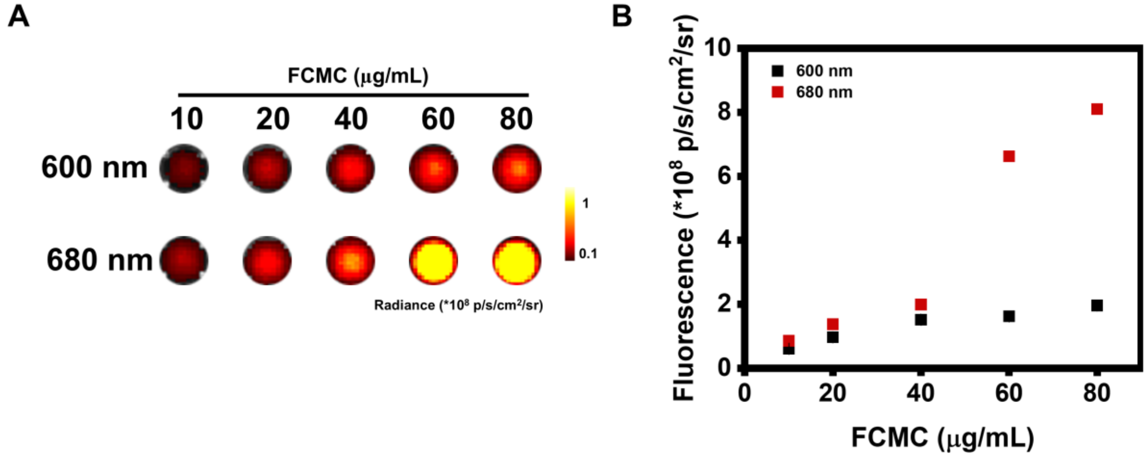

Figure S13. Fluorescence images at different concentrations of FCMC and statistics of fluorescence signal intensity (excitation at 500 nm).

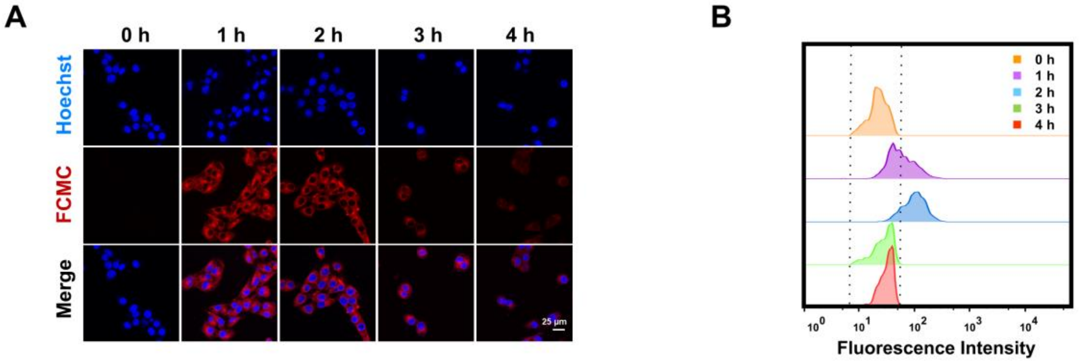

Figure S14. Cellular uptake behavior of FCMC was assessed by confocal laser scanning microscopy (**A**) and flow cytometry analysis (**B**).

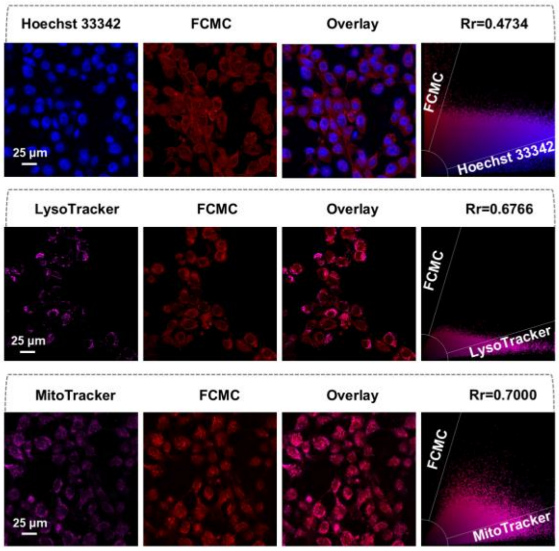

Figure S15. Subcellular colocalization assays of FCMC with commercial organelle dyes.

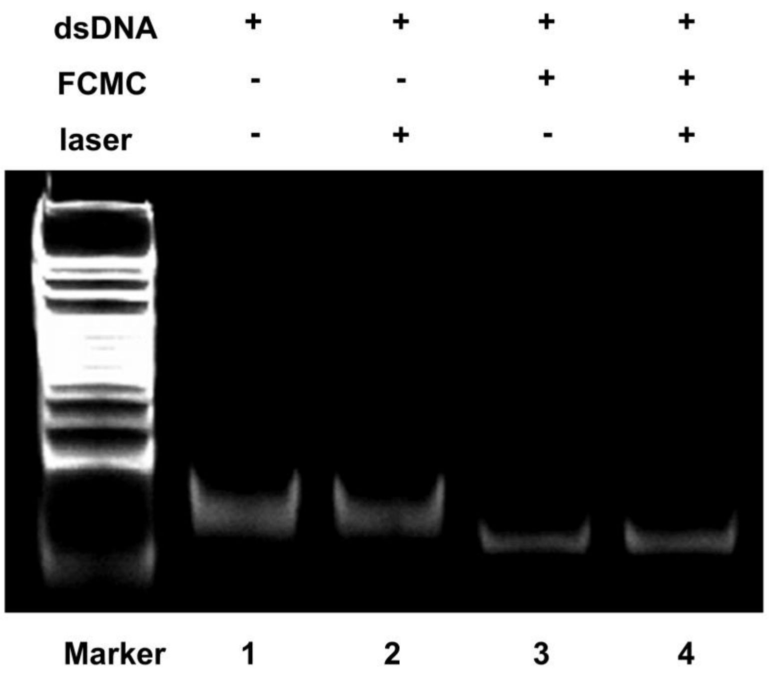

Figure S16. Polyacrylamide gel electrophoresis imaging of different solutions.

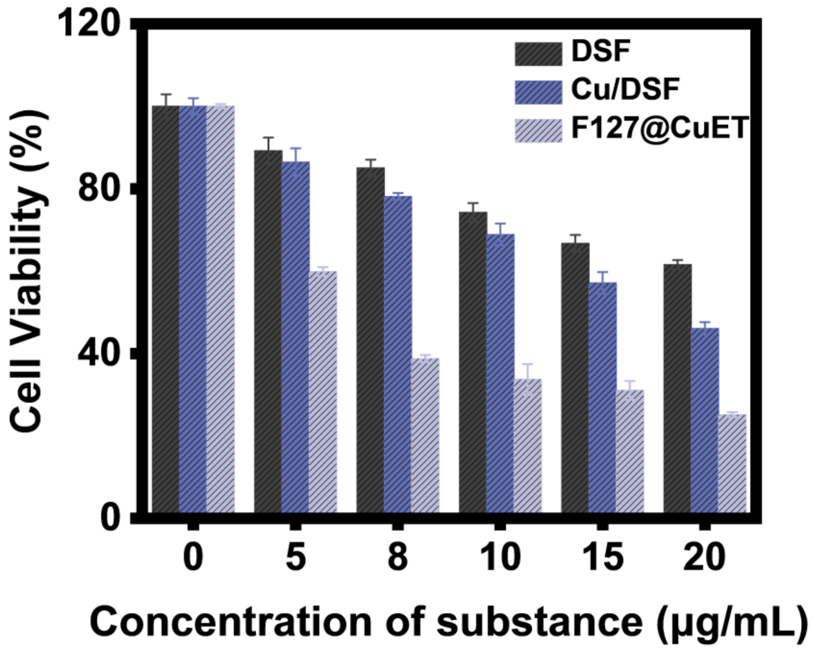

Figure S17. Cell viability of 4T1 cells after different treatments under various conditions for 24 h.

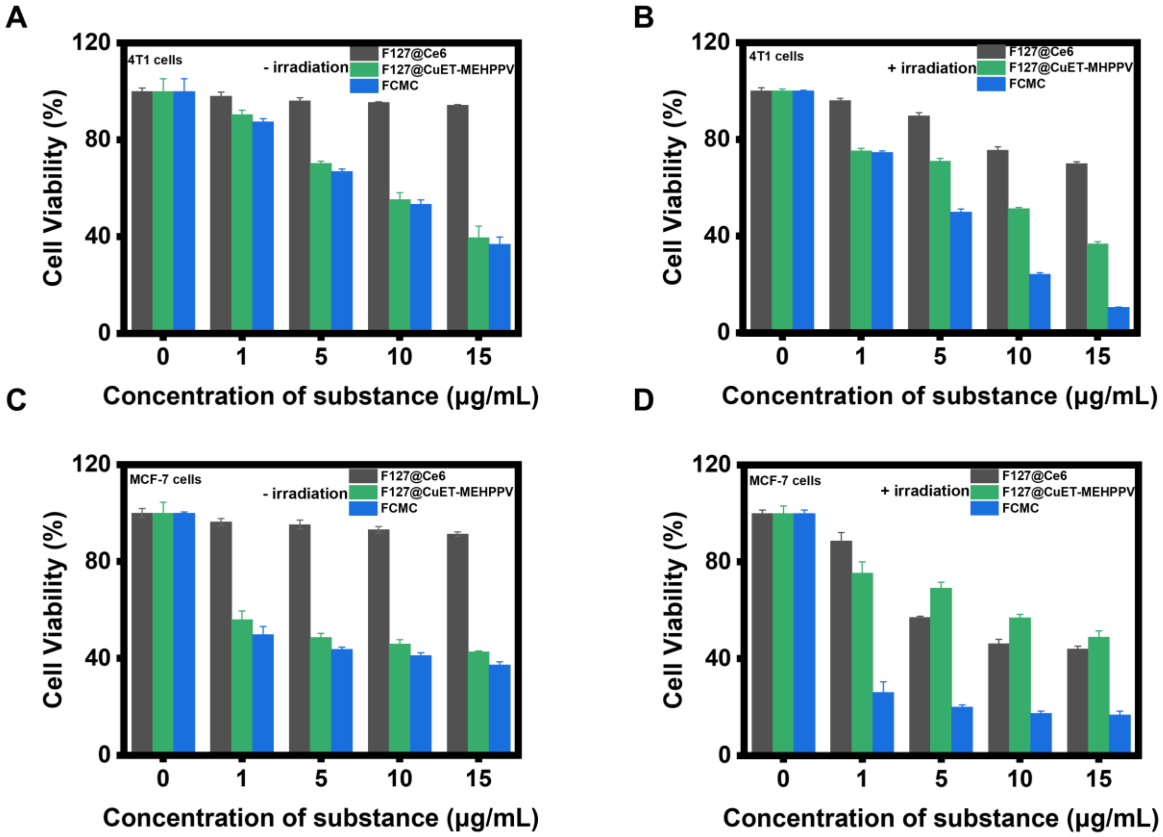

Figure S18. Cell viability of 4T1 cells (**A** and **B**) and MCF-7 (**C** and **D**) cells after different treatments under various conditions for 24 h.

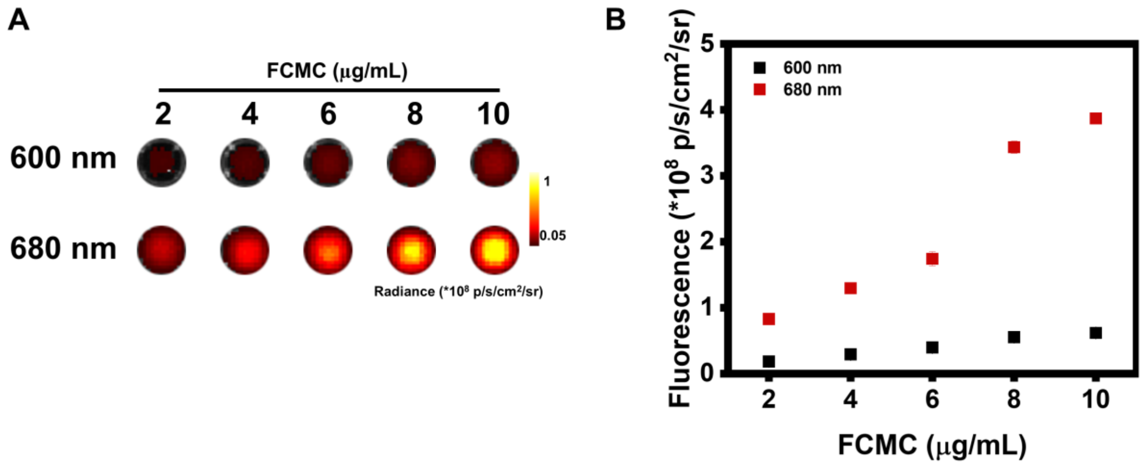

Figure S19. Fluorescence images at different concentrations of FCMC in cells and statistics of fluorescence signal intensity (excitation at 500 nm).

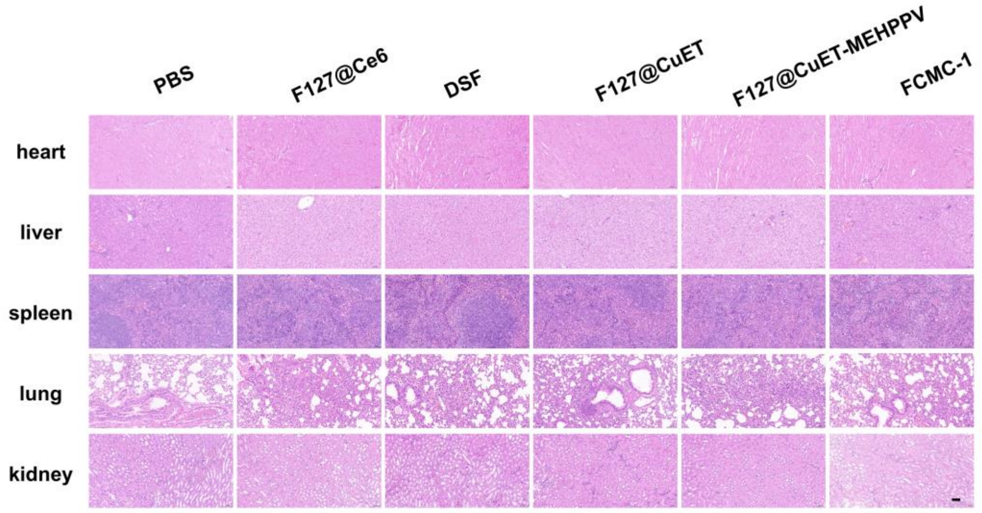

Figure S20. H&E staining images of the major organs after the different treatments without laser irradiation of 15 days. Scale bar, 50 μm.

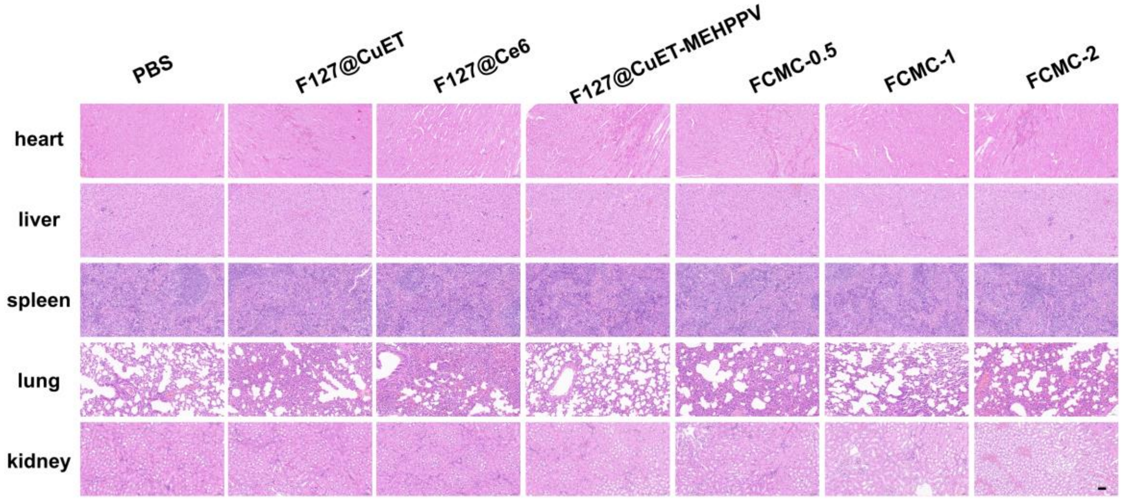

Figure S21. H&E staining images of the major organs after the different treatments with laser irradiation of 15 days. Scale bar, 50 μm.

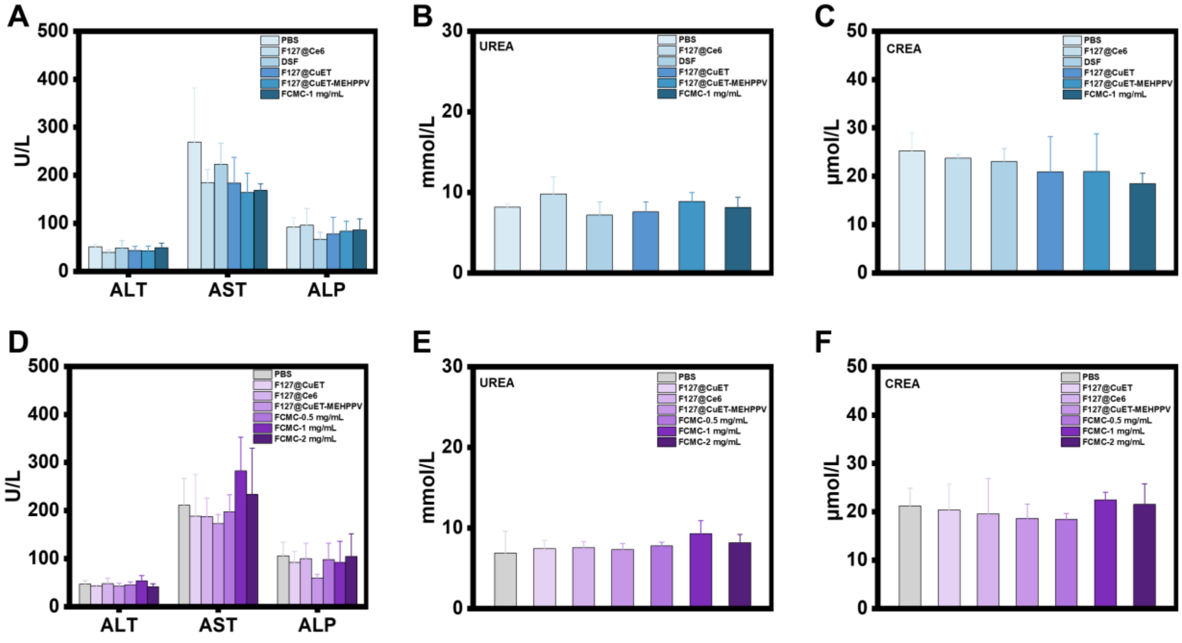

Figure S22. Blood biochemistry and hematology analysis.

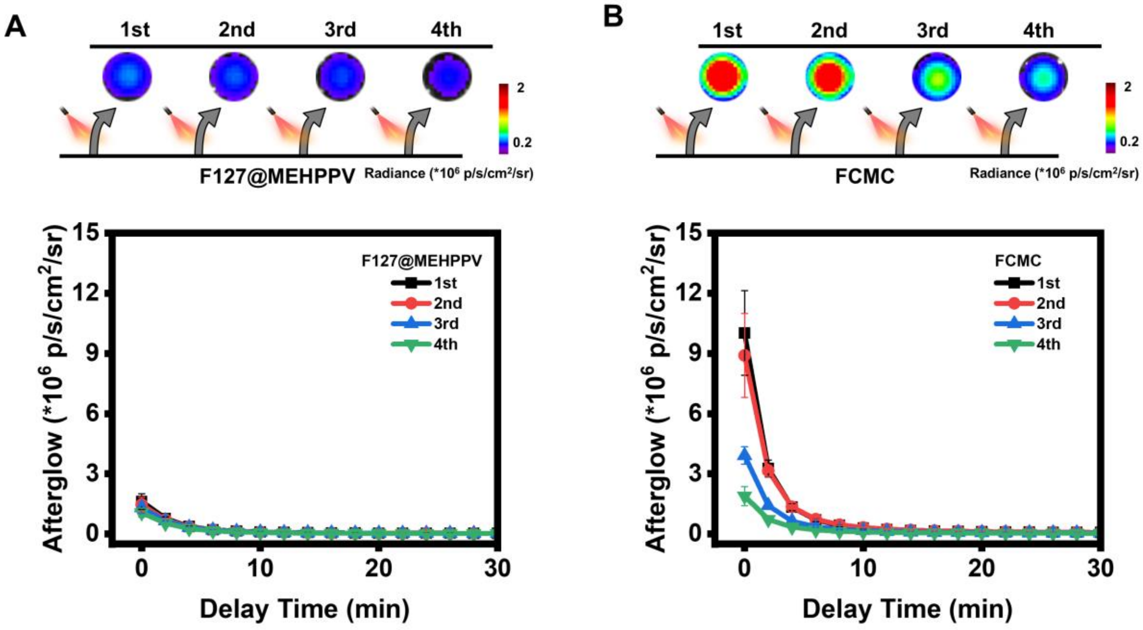

Figure S23. Afterglow images and quantification of afterglow intensities of (**A**) F127@MEHPPV and (**B**) FCMC after receiving different irradiation time.

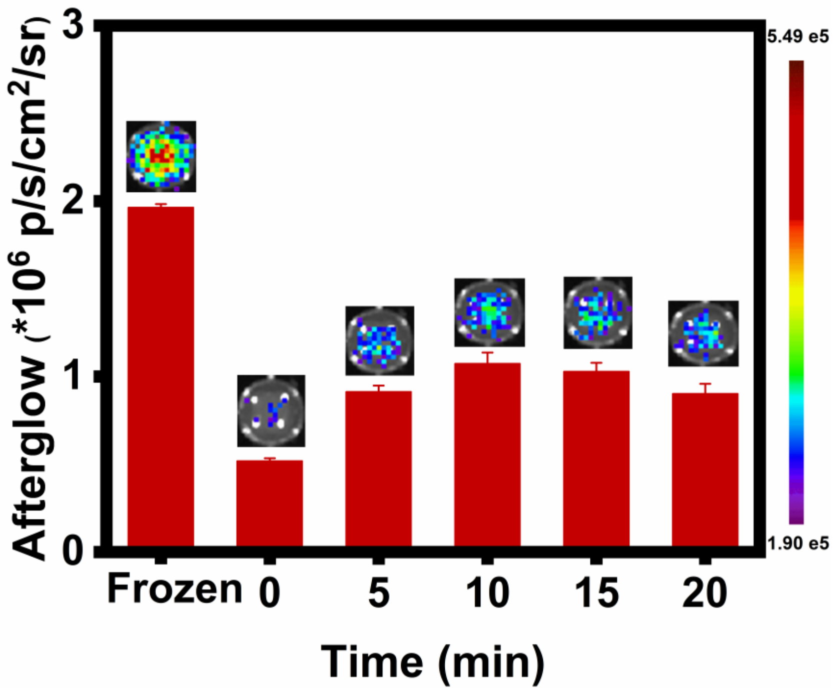

Figure S24. FCMC was pre-irradiated with a 650 nm laser (300 mW/cm^2) for 5 min followed by storage in -20 °C. Afterglow imaging and quantification of afterglow intensities was acquired after 24 h storage.

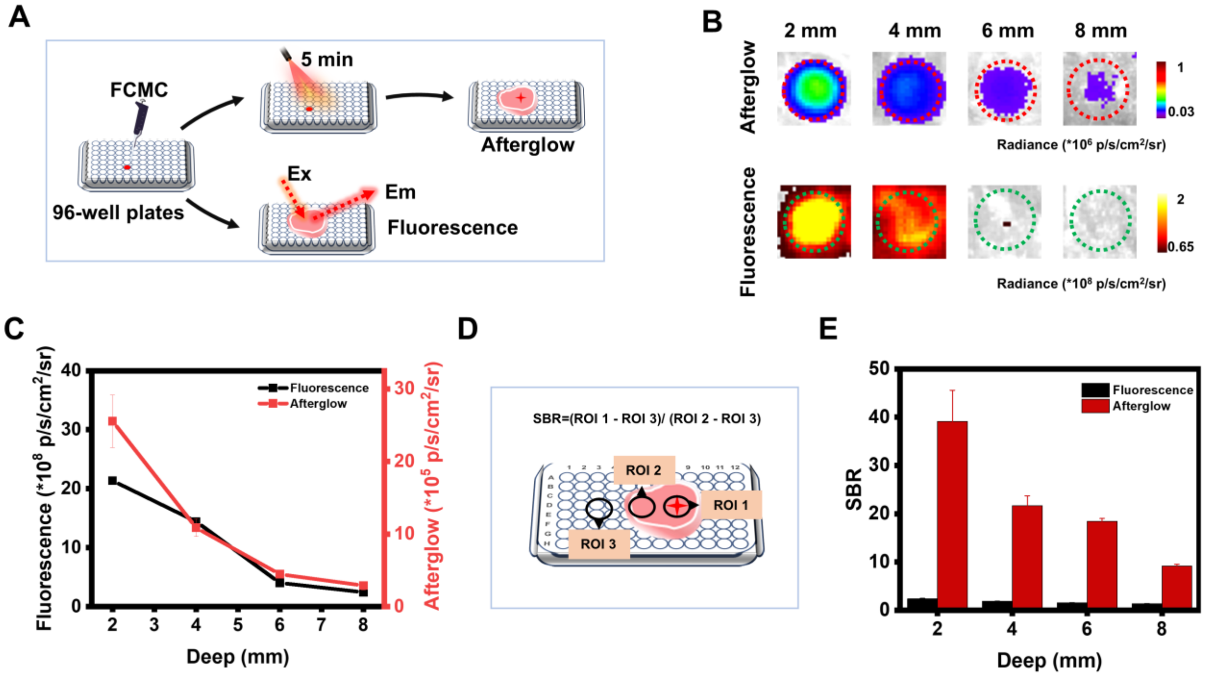

Figure S25. The scheme for tissue penetration study of FCMC (**A)**, afterglow luminescence and fluorescence imaging (**B)** and intensity (**C**). (**D** and **E**) SBR for afterglow luminescence and fluorescence of FCMC as a function of tissue depth.

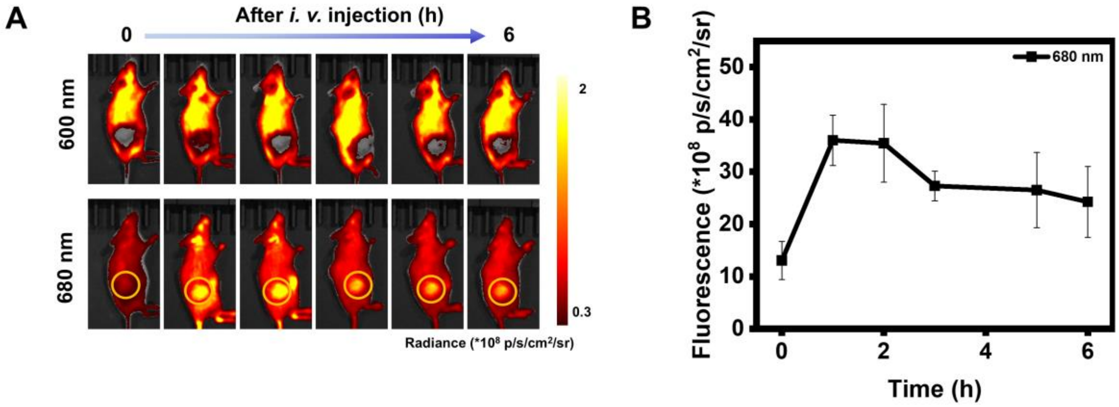

Figure S26. (**A**) Fluorescence imaging of the tumor site in mice at different time periods after *i.v.* injection of FCMC (2 mg/mL), with emission wavelengths of 600 nm and 680 nm. (**B**) statistics of fluorescence signal intensity.

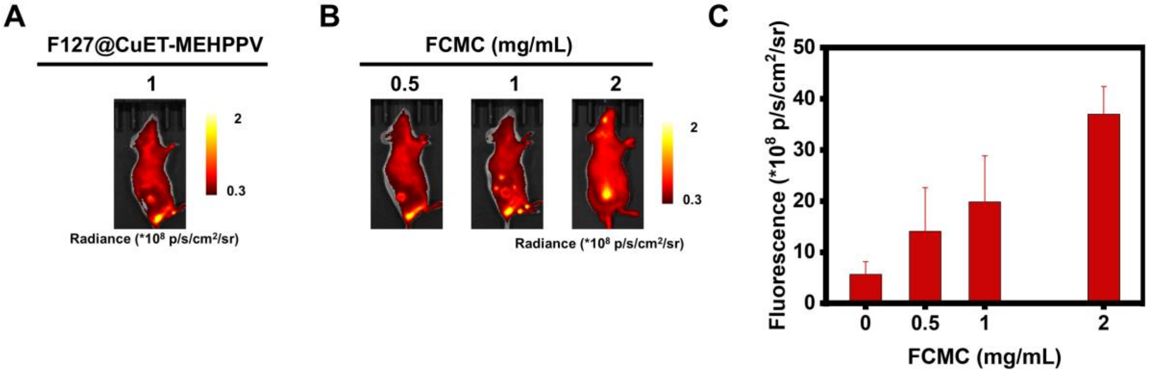

Figure S27. Fluorescence imaging of the tumor site after *i.v.* injection of (**A**) F127@CuET-MEHPPV (1 mg/mL) and (**B**) FCMC (0.5, 1, and 2 mg/mL) at an emission wavelength of 680 nm. (**C**) statistics of fluorescence signal intensity of the tumor site after *i.v.* injection of FCMC (0.5, 1, and 2 mg/mL).
